# A Topographic Atlas of the Human Brainstem in the Ponto-Mesencephalic Junction Plane

**DOI:** 10.3389/fnana.2021.627656

**Published:** 2021-08-13

**Authors:** Vincent Coulombe, Stephan Saikali, Laurent Goetz, Mohamad A. Takech, Éric Philippe, André Parent, Martin Parent

**Affiliations:** ^1^CERVO Brain Research Center, Quebec City, QC, Canada; ^2^Hôpital De L'Enfant-Jésus, CHU de Québec-Université Laval, Quebec City, QC, Canada; ^3^Hôpital Fondation Rothschild, Neurochirurgie pédiatrique - Unité Parkinson, Paris, France; ^4^Laboratoire d'Anatomie, Université Laval, Quebec City, QC, Canada

**Keywords:** midbrain, pons, medulla oblongata, cytoarchitecture, reticular formation, neurosurgery, neuropathology

## Abstract

The human brainstem harbors neuronal aggregates that ensure the maintenance of several vital functions. It also acts as a major relay structure for the neuronal information that travels between the cerebral cortex, the cerebellum and the spinal cord. As such, this relatively small portion of the human brain houses a multitude of ascending and descending fibers that course among numerous nuclei whose exact boundaries are still uncertain. Such a large number of nuclei and fiber tracts confined to a relatively small and compact brain region imposes upon the brainstem a highly complex cytoarchitectonic organization that still needs to be deciphered. The present work provides a topographic atlas of the human brainstem composed of 45 anatomical plates, each containing a pair of adjacent sections stained with Cresyl Violet and Luxol Fast Blue to help delineating brainstem nuclei and fiber tracts, respectively. The plates, which cover the entire midbrain, pons and medulla oblongata, are composed of equally-spaced sections referenced and aligned parallel to the ponto-mesencephalic junction rather than the fastigium or the obex. This topographic landmark is particularly suitable for neurosurgical interventions aiming at specific nuclei of the mesencephalic tegmentum. In complement, we provide 8 anatomical plates containing adjacent sections stained for choline acetyltransferase and Luxol Fast Blue, taken through the midbrain and the pons. This open access atlas of the human brainstem is intended to assist neuroanatomists, neurosurgeons and neuropathologists in their work.

## Introduction

The human brainstem plays a crucial role in the maintenance of vital functions, such as respiratory and cardiovascular activities. Furthermore, it acts as a major relay station between the cerebral cortex, the cerebellum and the spinal cord, as first suggested by the English neurologist Thomas Willis more than 350 years ago (Willis, 1664). Yet, despite its prime importance in the coordination of several basic central nervous system activities, the brainstem is one of the least understood parts of the human brain. The organizational complexity of the brainstem relies, at least in part, from the fact that its houses a multitude of ascending and descending fiber tracts that course among a large number of nuclei whose exact boundaries are still a matter of controversy. Such a large number of nuclei and fiber tracts restricted to a relatively small brain region [the brainstem occupies <3% of the total brain volume (Erbagci et al., 2012)] imposes upon the brainstem a highly complex cytoarchitectonic organization that still poses problems to neuroanatomists, neuropathologists, neurosurgeons, and imaging specialists.

Based on its external appearance along the rostrocaudal axis, the brainstem has traditionally been divided into the midbrain, the pons and the medulla oblongata. However, since the recent discovery of rhomobomeric segmentation based on developmental gene expression, the pons and medulla oblongata are now often referred together as the hindbrain, with the isthmus as its first segment (Watson et al., 2019; Paxinos et al., 2020). Along the dorsoventral axis, the brainstem can be divided into three distinctive parts: (1) a roof plate dorsal to the ventricular system known as the tectum, (2) a central core of cells and fibers beneath the ventricular system known as the tegmentum, and (3) a massive collection of ventrally located fibers derived from the cerebral cortex. The roof plate of the midbrain is represented by the tectum or quadrigeminal plate, consisting of the superior and inferior colliculi. At hindbrain levels, the roof plate is more elaborated and comprises the cerebellum and the tela choroidea, which will not be considered in detail here. The tegmentum of the midbrain and hindbrain contains the brainstem reticular formation or reticular core: a large collection of diffusely distributed cells closely intermingled with fibers that subserve multiple functions, and several more precisely delineated nuclei. The cortically derived ventral fiber system forms the crus cerebri at midbrain levels, one of the principal constituents of the ventral or basilar region at pontine levels, and the pyramids at medullary levels (Parent, 1996).

Some nuclei of the brainstem tegmentum are enriched in dopaminergic, cholinergic, serotoninergic or noradrenergic neurons known to be involved in the control of motor behavior, sleep and waking cycles, as well as various autonomic functions (Parent, 1996). Over the years, many efforts have been made to delineate brainstem nuclei and fiber tracts (Kolliker, 1896; Ziehen, 1903; Jacobsohn, 1909; Riley, 1943). Olszewski and Baxter were the first to provide a comprehensive and detailed description of the cytoarchitecture of the human brainstem illustrated with high-quality photomicrographs and outlined drawings of histological sections taken transversely to the longitudinal axis of the brainstem (Olszewski and Baxter, 1954). Despite the high quality of this work, the fact that no stereotaxic coordinates were provided has led many neurosurgeons to rely on the stereotaxic atlas of the human brain published by Schaltenbrand and Wahren (1977) for their interventions, with the inherent constraint of histological sections being oriented perpendicular to the anterior (ac) and posterior commissural (pc) plane. This coordinate system originally proposed by Talairach (Talairach et al., 1957; Talairach and Szikla, 1967; Talairach and Tournoux, 1988) is not ideally suited for brainstem stereotaxy because landmarks used for stereotaxic coordinates are too distant from regions of interest (Zrinzo et al., 2008; Goetz et al., 2019). The Allen Human Brain Atlas, which uses this sectioning plane, presents an overall view of brainstem structures in relation with the entire human brain by means of Nissl cell stain combined with parvalbumin and SMI-32 immunohistochemical markers (Ding et al., 2016). Efforts have also been deployed to provide brainstem stereotaxic atlases referenced on brainstem landmarks. Afshar et al. (1978), among others, have presented stereotaxic references for the human brainstem and cerebellar nuclei based on the floor of the fourth ventricle, the midline and a plane passing perpendicular to the floor of the fourth ventricle at the level of the fastigium. The stereotaxic atlas of Paxinos and Huang (1995) contains 64 histological sections perpendicular to the long axis of the brainstem, stained for Cresyl Violet and acetylcholine esterase as well as associated diagrams with delineated nuclei and fiber tracts. In their revised version (Paxinos et al., 2020), 159 plates are presented, 31 of which being from a different brain and used to delineate fiber tracts. In this atlas, sections of the brainstem were numbered based on their distance from the obex. More recently, numerous human brainstem descriptions from MRI have been published (Naidich et al., 2007; Deistung et al., 2013; Bianciardi et al., 2015; Tang et al., 2018; Rushmore et al., 2020). Among these, the Duvernoy's non-stereotaxic atlas of the human brainstem correlates transverse histological brainstem sections with corresponding clinical 3T and 9.4T MRI (Naidich et al., 2007) and is still widely used. The recent work of Rushmore and collaborators offers a detailed map of the human brainstem based on MRI dataset composed of 50-micron isotropic voxels from a post-mortem human brain (Rushmore et al., 2020).

In more recent studies (Ferraye et al., 2010; Goetz et al., 2019), the use of a new coordinate system referenced upon the ponto-mesencephalic junction (PMJ, a line that connects the inferior aspect of the quadrigeminal plate posteriorly with the foramen caecum of the interpeduncular fossa anteriorly), the floor of the fourth ventricle and the lateral mesencephalic sulci has been suggested to be more suitable for brainstem stereotaxy. This coordinate system, which refers to mesencephalic landmarks rather than the fastigium or the obex, has been adopted by some research groups (Thevathasan et al., 2011, 2012; Insola et al., 2012). It appears to be particularly suitable for neurosurgical interventions in the mesencephalic reticular formation, mainly because the references used are closer to neurosurgical areas of interest and easy to identify from MRI (Zrinzo et al., 2008). Readers looking for a color atlas of brainstem surgery should refer to the work of Spetzler and collaborators (Spetzler et al., 2017).

Our long-term interest in the basic aspects of the pathogenesis of various neurodegenerative diseases for which patients often present brainstem anomalies or are candidate for brainstem surgical interventions in the case of Parkinson's disease, has led us to produce a topographic atlas of the human brainstem composed of 45 anatomical plates, each containing a pair of adjacent sections stained with Cresyl Violet and Luxol Fast Blue used to delineate brainstem nuclei and fiber tracts, respectively. The plates cover the midbrain, the pons and the medulla oblongata and are composed of transverse sections taken parallel to the PMJ. In complement, we provide eight anatomical plates containing adjacent sections stained for choline acetyltransferase (ChAT) and Luxol Fast Blue taken through the midbrain and the pons. We hope that this open access atlas of the human brainstem will complement the work of our predecessors while providing an additional tool for fundamental and clinical research.

## Method

### Specimen Preparation

**Figure 1:**
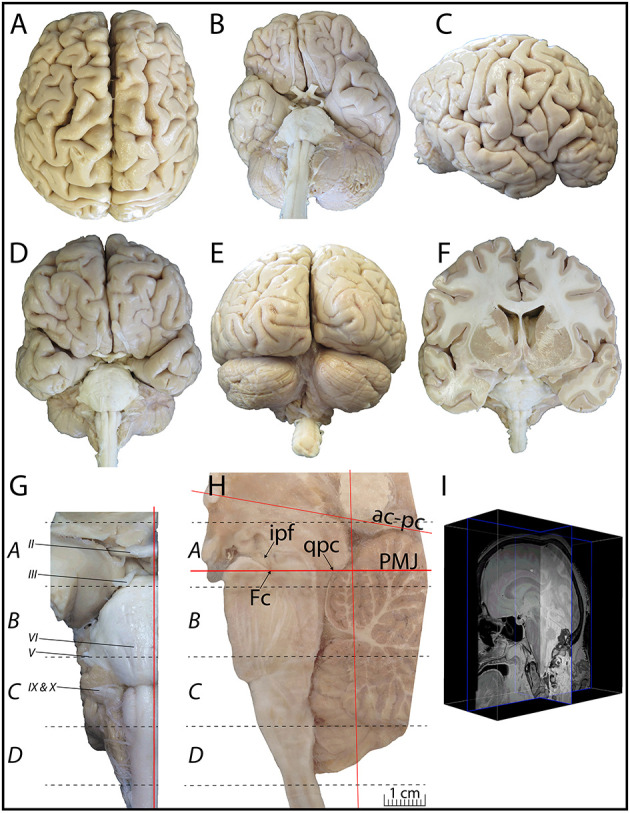
**(A–E)** Superior **(A)**, inferior **(B)**, lateral **(C)**, anterior **(D)**, and posterior **(E)** views of the whole brain used. This brain comes from a generous donation of a 61-year-old woman who died from a pluri-metastatic colorectal cancer with no signs of neurological, neurodegenerative or psychiatric diseases. The perfused and extracted brain weighed 1,328 g. **(F)** Coronal view of the sectioned brain. **(G)** Picture of the dissected brainstem cut through its longitudinal axis, along a sagittal plane, at 1 mm from the midline. **(H)** From the right hemi-brainstem containing the midline, four blocks were cut along the ponto-mesencephalic junction plane (PMJ in **H**). Block A extends from 11.19 to −0.98 mm (plates 1–11), block B from −3.87 to −21.33 mm (plates 12–24), block C from −24.09 to −36.87 mm (plates 25–38), and block D from −39.70 to −51.70 mm (plates 39–45), relative to the PMJ. **(I)** Brain MRI is available as Supplementary Material.

The post-mortem material was gathered from the brain of a 61-year-old woman who died from a pluri-metastatic colorectal cancer with no signs of neurological, neurodegenerative or psychiatric diseases. After a post-mortem delay of 5.5 h, the brain was perfused through the internal carotids and the vertebral arteries with 4 L of cold saline solution (NaCL 0.9% to which 20 units/mL of heparin was added), followed by 6 L of a cold fixative solution containing 4% paraformaldehyde (PFA) diluted in phosphate buffer (PB, 0.1 M, pH 7.4) and 4 L of 4% PFA to which 0.3% of glutaraldehyde was added. We then performed a MRI scan of the head with a 3T Philips Achieva (TE: 17 ms; TR:4000 ms; slice thickness: 500 μm; duration 8 h, see Supplementary Material) before brain extraction. The extracted brain weighed 1,328 g (Figures 1A–F). The brainstem was dissected out and post-fixed by immersion in 4% PFA for 4 days. Tissue samples were obtained from the Human Anatomy Laboratory at Université Laval and kept in the Human Brain Bank of the CERVO research Center. Both Institutions required informed consent before donation of tissues. The Ethics Committee at Université Laval approved the brain collecting procedures, as well as the storage and handling of post-mortem human brain tissues.

The brainstem was dissected and cut along a sagittal plane at 1 mm from the midline (Figure 1G). From the right hemi-brainstem that contained the midline, four different blocks were cut along the ponto-mesencephalic junction plane (PMJ), a line that connects the inferior aspect of the quadrigeminal plate (qpc) posteriorly with the foramen caecum (Fc) of the interpeduncular fossa (ipf) anteriorly (Figure 1H). The PMJ is depicted by a red line on the different plates. Its exact position can be found on Figure 1H and on plate 10 (0.00 mm). We estimate that there exists a difference of 10 degrees between the PMJ plane and the ac-pc plane (Figure 1H) and 20 degrees between the PMJ plane and the one used in the atlas of Paxinos (Paxinos et al., 2020). Each of the blocks were then cut at 4°C into 50 μm-thick sections using a vibratome (Leica VT1200S). The sections were all collected serially in phosphate buffer saline (PBS, 0.1 M, pH 7.4). Pairs of adjacent sections were selected out of 22 sections and then processed for Cresyl Violet and Luxol Fast Blue, respectively, providing a mean interval of 1.1 mm between each plate. Because some sections were damaged during the cutting process, in some cases, we decided to choose the next undamaged section instead of the adjacent one, for a maximum of 400 μm interval between Cresyl Violet and Luxol Fast Blue stained sections. Because losses of tissue inevitably occurred between each block, in addition to the known section thickness and intervals, we referred to the MRI in order to confirm the exact position of each section, relative to the PMJ (Figure 1I).

### Cresyl Violet Staining

Sections intended for Cresyl Violet staining were first mounted on gelatin-coated microscope slides, air dried at room temperature and incubated overnight in 95% ethanol at 56°C. The slides were then rinsed 15 times in distilled water and immersed for 3 min at room temperature in a pre-heated and filtered solution of Cresyl Violet acetate (catalog no. C5042, Sigma, St-Louis, MO, USA) dissolved at 0.1% in distilled water. Sections were then rinsed in distilled water followed by 10 times in ethanol 95%, two times in a solution of ethanol/acid acetic 0.5%, five times in ethanol 95%. The rinses in ethanol 95% and ethanol/acid acetic were repeated until the desired staining intensity was obtained. Sections were dehydrated in ethanol and coverslipped with Permount (Permount Mounting Medium, catalog no. SP15, Thermo Fisher Scientific, Waltman, MA, USA).

### Luxol Fast Blue Staining

Sections intended for Luxol Fast Blue staining were first mounted on gelatin-coated microscope slides, air dried at room temperature and rinsed 10 times in ethanol 95%. They were then incubated overnight at 56°C in Luxol Fast Blue (Solvent Blue 38, catalog no. S3382, Sigma, St-Louis, MO, USA) dissolved at 0.1% in ethanol. They were rinsed once in ethanol 95%, 10 times in lithium carbonate (0.01%) and 10 times in ethanol 70%. When needed, rinses in lithium carbonate (0.01–0.1%) and ethanol were repeated until the desired staining intensity was obtained. Sections were dehydrated in ethanol and coverslipped with Permount. To increase contrast, five sections stained for Luxol Fast Blue had to be also stained with Cresyl Violet (Plates 9, 10, 26, 31, 37).

### Choline Acetyltransferase Immunostaining

Eight additional equally-spaced sections taken trough the midbrain and the pons (between +4.88 mm and −10.59 mm) were stained for choline acetyltransferase (ChAT), the enzyme responsible for the synthesis of acetylcholine (plates A-H). After three rinses in PBS, the sections were placed for 20 min at room temperature in hydrogen peroxide (3% H_2_O_2_ diluted in ethanol) to eliminate endogenous peroxidase activity. The free-floating sections were then placed in sodium borohydride (0.5% diluted in PBS) for 30 min. After three rinses in PBS, the sections were preincubated for 1 h at room temperature in a PBS solution containing 2% normal rabbit serum and 0.5% Triton X-100, and incubated 48 h at 4°C in the same solution to which the goat anti-ChAT antibody was added (catalog no. AB144P, EMD Milipore Corporation, Billerica, MA, 1:20). The sections were then rinsed, reincubated for 1 h at room temperature with a rabbit anti-goat biotinylated IgG (catalog # BA-5000; Vector Labs, Burlingame, CA, USA; 1:200). After three rinses in PBS, the sections were reincubated for 1 h at room temperature in 2% avidin-biotin-peroxidase complex (catalog # PK-4000; Vector Labs). The bound peroxidase was revealed by placing the sections in a medium containing 0.05% 3,3′-diaminobenzidine tetrahydrochloride (DAB, catalog #D5637; Sigma) and 0.005% H_2_O_2_ in 0.05 M Tris buffer, pH 7.6. The reaction was stopped after 8 min by several washes in Tris buffer and PBS. Immunostained sections were mounted on gelatine-coated slides, dehydrated in ethanol and coverslipped with Permount.

Cresyl Violet, Luxol Fast Blue and ChAT-stained sections were digitalized at 1200 dpi (pixel resolution of 1 μm) using a slide scanner (TISSUEscopeTM 4000, Huron Technologies, Waterloo, Ontario, Canada) equipped with a 10X objective.

### Methodological Considerations and Limitations

Chemical fixation of the brain and section processing inevitably lead to shrinkage. In order to minimize shrinkage, blocks were cut at 4°C with a vibratome, precluding the need of cryoprotection in sucrose solution and freezing. A comparison of the size of the red nucleus as it appears on post-mortem brain MRI images and on Cresyl Violet stained sections, has revealed the shrinkage to be of the order of 4%. No pre-mortem MRI was available to assess shrinkage that might have been caused by the perfusion. It should be reminded that this atlas is based on a single brain of a 61-year-old woman and that existing inter-individual variations should carefully be taken into account using provided MRI (see Supplementary Material). Three different magnifications had to be used to provide plates presenting histological sections of adequate size (plates 1–6, plates 7–29, and plates 30–45). Therefore, readers should pay close attention to individual scale bars when comparisons are made between plates. Segmentation of brainstem nuclei and fiber tracts from Cresyl Violet and Luxol Fast Blue stained sections were performed following careful examination of histological sections, so as to avoid arbitrary delineation. Only structures that could be easily delineated in our preparations are identified and dashed lines are used when brainstem regions don't show clear histological boundaries. For example, the cuneiform nucleus (CnF) and the tegmental part of pontine reticular nucleus (PnTn) are broadly delineated with dashed lines because their boundaries with surrounding structures could not be clearly established. Therefore, the CnF, as defined in the present study, might comprise a portion of the mesencephalic reticular formation, whereas the PnTn could include parts of the isthmic reticular formation, the retroisthmic nucleus, the retrorubral field and the ventrolateral tegmental nucleus, as defined by Paxinos (Paxinos et al., 2020). Our segmentation was confirmed with the help of other human brainstem atlases (Olszewski and Baxter, 1954; Schaltenbrand and Wahren, 1977; Afshar et al., 1978; Paxinos and Huang, 1995; Naidich et al., 2007; Paxinos et al., 2020). Readers might refer to other human brainstem atlases for more extensive delineation of brainstem subnuclei and additional plates, in different sectioning planes. The nomenclature and abbreviations used in the present study are largely based on those of Paxinos and Watson (1982). For brainstem nuclei that didn't show clear subdivisions, we used single abbreviations to identify structures composed of several subnuclei. For example, the abbreviation Sp5 for spinal trigeminal nucleus, as delineated in the present atlas, includes the oral (Sp5O), interpolar (Sp5I), and caudal (Sp5C) parts of the nucleus, and the abbreviation VC for ventral cochlear nucleus includes the anterior (VCA) and posterior (VCP) parts of the nucleus that are delineated in other atlases. Likewise, the abbreviation PAG for periaqueductal gray refers to all PAG columns. A complete list of abbreviations and equivalent Latin names, as published in Terminologia Anatomica (Terminology, 1998) is also provided. The asterisks added to some abbreviations indicate that these abbreviations are slightly different from those used in the human brainstem atlas of Paxinos (Paxinos et al., 2020) or point to structures that are not identified in that atlas.

**Table:** **LIST OF ABBREVIATIONS**

3N	Oculomotor nucleus	*Nucleus nervi oculomotorii*
3n	Oculomotor nerve	*Nervus oculomotorius*
4N	Trochlear nucleus	*Nucleus nervi trochlearis*
4n	Trochlear nerve	*Nervus trochlearis*
4V	Fourth ventricle	*Ventriculus quartus*
5ADi	Motor trigeminal nucleus, anterior digastric part	*Nucleus motorius nervi trigemini, pars digastrica anterior*
5N	Motor trigeminal nucleus	*Nucleus motorius nervi trigemini*
5Sp	Lamina 5 of the spinal gray	*Lamina spinalis V*
5Te	Motor trigeminal nucleus, temporal part	*Nucleus motorius nervi trigemini, pars temporalis*
5Tr	Trigeminal transition zone	*Zona transitionalis nervi trigemini*
6N	Abducens nucleus	*Nucleus nervi abducentis*
6n	Abducens nerve	*Nervus abducens*
7DM	Facial nucleus, dorsomedial part	*Nucleus nervi facialis, pars dorsomedialis*
7N	Facial nucleus	*Nucleus nervi facialis*
7n	Facial nerve	*Nervus facialis*
7SH	Facial nucleus, stylohyoid part	*Nucleus nervi facialis, pars stylohyoidalis*
7VL	Facial nucleus, ventrolateral part	*Nucleus nervi facialis, pars ventrolateralis*
8n	Vestibulocochlear nerve	*Nervus vestibulocochlearus*
9n	Glossopharyngeal nerve	*Nervus glossopharyngeus*
9Sp	Lamina 9 of the spinal gray	*Lamina spinalis IX*
10N	Dorsal motor nucleus of the vagus nerve	*Nucleus dorsalis motorius nervi vagi*
10n	Vagus nerve	*Nervus vagus*
12N	Hypoglossal nucleus	*Nucleus nervi hypoglossi*
12n	Hypoglossal nerve	*Nervus hypoglossus*
ac^*^	Anterior commissure	*Commissura anterior*
al^*^	Ansa lenticularis	*Ansa lenticularis*
Amb	Ambiguus nucleus	*Nucleus ambiguous*
Amg^*^	Amygdaloid complex	*Corpus amygdaloideum*
Aq	Aqueduct	*Aqueductus cerebri*
Ar	Arcuate nucleus	*Nucleus arcuatus*
BIC	Nucleus of the brachium of the inferior colliculus	*Nucleus brachium colliculi inferioris*
bic	Brachium of the inferior colliculus	*Brachium colliculi inferioris*
CAT	Nucleus of the central acoustic tract	*Nucleus centralis tractus acustica*
CbH^*^	Cerebellar hemisphere	*Hemispherium cerebelli*
CbV^*^	Cerebellar vermis	*Vermis cerebelli*
CC	Central canal	*Canalis centralis*
Cd^*^	Caudate nucleus	*Nucleus caudatus*
CG^*^	Central gray	*Griseum centralis*
CLi	Caudal linear nucleus of the raphe	*Nucleus raphes linearis, pars caudalis*
CnF	Cuneiform nucleus	*Nucleus cuneiformis*
cp	Cerebral peduncle	*Pedunculus cerebri*
csp	Corticospinal tract	*Tractus corticospinalis*
Ct	Conterminal nucleus	*Nucleus conterminalis*
ctg	Central tegmental tract	*Tractus tegmentalis centralis*
cth^*^	Cerebellothalamic tract	*Tractus cerebellothalamicus*
Cu	Cuneate nucleus	*Nucleus cuneatus*
cu	Cuneate fasciculus	*Fasciculus cuneatus*
DC	Dorsal cochlear nucleus	*Nucleus cochlearis dorsalis*
DLG	Dorsal lateral geniculate nucleus	*Nucleus geniculatum laterale*
DLL	Dorsal nucleus of the lateral lemnicus	*Nucleus dorsalis lemnisci lateralis*
DPGi	Dorsal paragigantocellular nucleus	*Nucleus paragigantocellularis dorsalis*
DR	Dorsal raphe nucleus	*Nucleus raphes dorsalis*
dsc	Dorsal spinocerebellar tract	*Tractus spinocerebellaris dorsalis*
DTg^*^	Dorsal tegmental nucleus	*Nucleus tegmentalis dorsalis*
ECu	External cuneate nucleus	*Nucleus cuneatus, pars externa*
emlt^*^	External medullary lamina of the thalamus	*Lamina medullaris externa thalami*
EnR	Endorestiform nucleus	*Nucleus endorestiformis*
EW	Edinher-Westphal nucleus	*Nucleus accessorii nervi oculomotorii*
Fc^*^	Foramen caecum	*Foramen caecum*
ft^*^	Thalamic fasciculus	*Fasciculus thalamicus*
fx^*^	Fornix	*Fornix*
GP^*^	Globus pallidus	*Globus pallidus*
Gr	Gracile nucleus	*Nucleus gracilis*
gr	Gracile fasciculus	*Fasciculus gracilis*
I8	Interstitial nucleus of the vestibular part of the 8th nerve	*Nucleus interstitialis nervi vestibulocochlearus, pars vestibularis*
ia	Internal arcuate fibers	*Fibrae arcuatae internae*
IC^*^	Inferior colliculus	*Colliculi inferioris*
ic^*^	Internal capsule	*Capsula interna*
icp	Inferior cerebellar peduncle	*Pedunculus cerebellaris inferior*
IF12^*^	Interfascicular nucleus of the hypoglossal nerve	*Nucleus interfascicularis nervi hypoglossi*
InC	Interstitial nucleus of Cajal	*Nucleus interstitialis*
IOD	Inferior olive, dorsal nucleus	*Nucleus olivaris dorsalis*
IOM	Inferior olive, medial nucleus	*Oliva inferior, nucleus medialis*
IOPr	Inferior olive, principal nucleus	*Oliva inferior, nucleus principalis*
IP	Interpeduncular nucleus	*Nucleus interpeduncularis*
ipf	Interpeduncular fossa	*Fossa interpeduncularis*
JxO	Juxtaolivary nucleus	*Oliva inferior, juxta nucleus*
LC	Locus coeruleus	*Locus coeruleus*
lcs	Lateral corticospinal tract	*Tractus corticospinalis lateralis*
LDTg	Laterodorsal tegmental nucleus	*Nucleus tegmentalis laterodorsalis*
Li	Linear nucleus of the hindbrain	*Nucleus linearis rhombencephali*
ll	Lateral lemniscus	*Lemniscus lateralis*
LPB	Lateral parabrachial nucleus	*Nucleus parabrachialis lateralis*
LPCu	Lateral pericuneate nucleus	*Nucleus pericuneatus lateralis*
LPGi	Lateral paragigantocellular nucleus	*Nucleus paragigantocellularis lateralis*
LRt	Lateral reticular nucleus	*Nucleus reticularis lateralis*
LRtS5	Lateral reticular nucleus, subtrigeminal part	*Nucleus reticularis lateralis, pars subtrigeminalis*
LVe	Lateral vestibular nucleus	*Nucleus vestibularis lateralis*
m5	Motor root of the trigeminal nerve	*Radix motoria nervus trigeminus*
MB	Mammillary body	*Corpus mammillare*
mcp	Middle cerebellar peduncle	*Pedunculus cerebellaris medius*
MdC^*^	Medullary reticular nucleus, central part	*Formatio reticularis medulares, pars centralis*
MdD	Medullary reticular nucleus, dorsal part	*Formatio reticularis medulares, pars dorsalis*
MdV	Medullary reticular nucleus, ventral part	*Formatio reticularis medulares, pars ventralis*
Me5	Mesencephalic trigeminal nucleus	*Nucleus mesencephalicus nervi trigemini*
me5	Mesencephalic trigeminal tract	*Tractus mesencephalici nervi trigemini*
MG	Medial geniculate nucleus	*Nucleus geniculatum mediale*
ml	Medial lemniscus	*Lemniscus medialis*
mlf	Medial longitudinal fasciculus	*Fasciculus longitudinalis medialis*
MnR	Median raphe nucleus	*Nucleus raphes medianus*
MPB	Medial parabrachial nucleus	*Nucleus parabrachialis medialis*
MPCu	Medial pericuneate nucleus	*Nucleus pericuneatus medialis*
mt^*^	Mammillothalamic tract	*Tractus mammillothalamicus*
MVe	Medial vestibular nucleus	*Nucleus vestibularis medialis*
NB	Basal nucleus of Meynert	*Nucleus basalis Meynerti*
oc	Olivocerebellar tract	*Tractus olivocerebellaris*
opt	Optic tract	*Tractus opticus*
P5	Peritrigeminal zone	*Zona peritrigeminalis*
PAG	Periaqueductal gray	*Substantia grisea centralis*
PBP	Parabrachial pigmented nucleus of the ventral tegmental area	*Nucleus pigmentosus parabrachialis*
pc	Posterior commissure	*Commissura posterior*
PDTg	Posterodorsal tegmental nucleus	*Nucleus tegmentalis posterodorsalis*
PN	Paranigral nucleus of the ventral tegmental area	*Nucleus paranigralis*
Pn	Pontine nuclei	*Nuclei pontis*
PnB	Pontobulbar nucleus	*Nucleus pontobulbaris*
PnC	Pontine reticular nucleus, caudal part	*Formatio reticularis pontis, pars caudalis*
PnG^*^	Pontine reticular nucleus, gigantocellular part	*Formatio reticularis pontis, pars gigantocellularis*
PnO	Pontine reticular nucleus, oral part	*Formatio reticularis pontis, pars oralis*
PnP^*^	Pontine reticular nucleus, parvocellular part	*Formatio reticularis pontis, pars parvocellularis*
PnR	Pontine raphe nucleus	*Nucleus raphes pontis*
PnTn^*^	Pontine reticular nucleus, tegmental part	*Formatio reticularis pontis, pars tegmentalis*
PP	Peripeduncular nucleus	*Nucleus peripeduncularis*
Pr	Prepositus nucleus	*Nucleus prepositus*
Pr5	Principal sensory trigeminal nucleus	*Nucleus principalis nervi trigemini*
PT^*^	Pretectal nucleus	*Nucleus pretectalis*
PTg	Pedunculotegmental nucleus	*Nucleus pedunculotegmentalis*
Pul	Pulvinar nucleus	*Nuclei pulvinares*
Put^*^	Putamen	*Putamen*
py	Pyramidal tract	*Tractus pyramidalis*
pyx	Pyramidal decussation	*Decussatio pyramidum*
R^*^	Red nucleus	*Nucleus ruber*
RAmb	Retroambiguus nucleus	*Nucleus retroambiguus*
RIP	Raphe interpositus nucleus	*Nucleus raphes interpositus*
RMg	Raphe magnus nucleus	*Nucleus raphes magnus*
ROb	Raphe obscurus nucleus	*Nucleus raphes obscurus*
RtTg	Reticulotegmental nucleus	*Nucleus reticularis tegmenti*
s5	Sensory root of the trigeminal nerve	*Radix sensoria nervus trigeminus*
SC	Superior colliculus	*Colliculus superioris*
scp	Superior cerebellar peduncle	*Pedunculus cerebellaris superior*
SGe	Supragenual nucleus of the raphe	*Nucleus raphes supragenualis*
SNC	Substantia nigra, compact part	*Nucleus substantia nigra, pars compacta*
SNR	Substantia nigra, reticular part	*Nucleus substantia nigra, pars reticulata*
SOl	Superior olive	*Oliva superior*
Sol	Solitary nucleus	*Nucleus solitarius*
sol	Solitary tract	*Tractus solitarii*
Sp5^*^	Spinal trigeminal nucleus	*Nucleus spinalis nervi trigemini*
sp5	Spinal trigeminal tract	*Tractus spinalis nervi trigemini*
SpVe	Spinal vestibular nucleus	*Nucleus vestibularis*
STh	Subthalamic nucleus	*Nucleus subthalamicus*
SubC^*^	Subcoeruleus nucleus	*Nucleus subcoeruleus*
SuL-B9^*^	Supralemniscal nucleus–B9 serotonin cells	*Nucleus supralemniscalis*–*B9*
SuVe	Superior vestibular nucleus	*Nucleus vestibularis superior*
tfp	Transverse fibers of the pons	*Fibrae pontis transversae*
VC^*^	Ventral cochlear nucleus	*Nucleus cochlearis ventralis*
vesp	Vestibulospinal tract	*Tractus vestibulospinalis*
VLL	Ventral nucleus of the lateral lemniscus	*Nucleus ventralis lemnisci lateralis*
vsc	Ventral spinocerebellar tract	*Tractus spinocerebellaris ventralis*
VTA	Ventral tegmental area	*Area tegmentalis ventralis*
xscp	Decussation of the superior cerebellar peduncle	*Decussatio pedunculorum cerebellarium superiorum*

* *Asterisks indicate abbreviations that are slightly different from those used in the human brainstem atlas of Paxinos et al. (2020) or point to structures that are not identified in that atlas*.

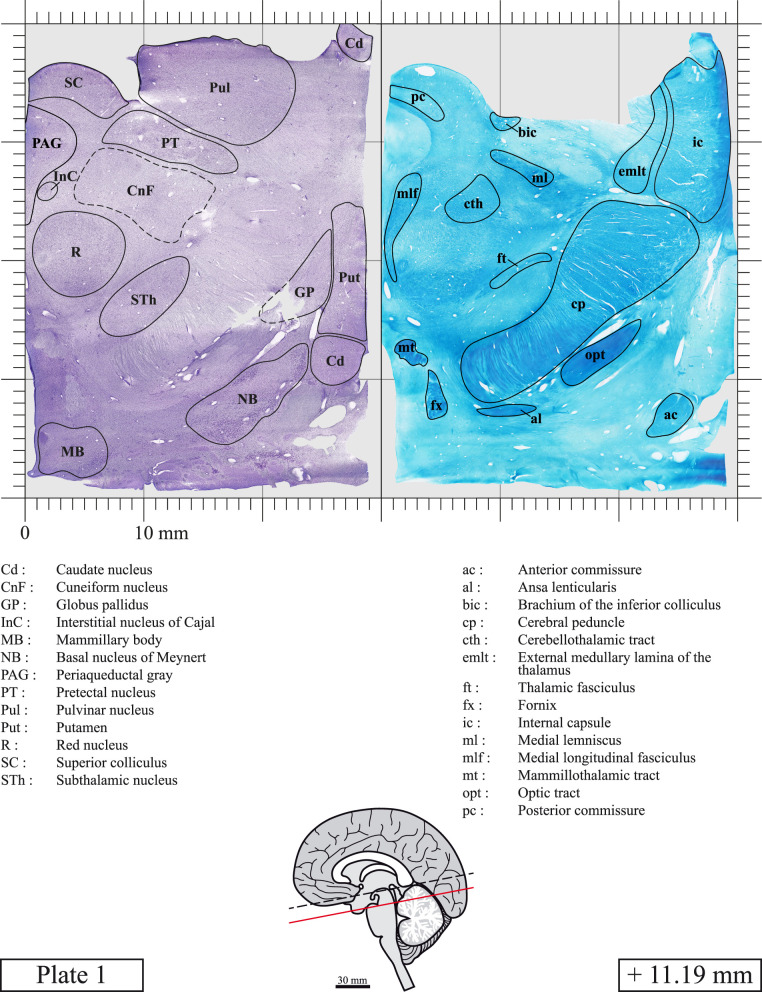

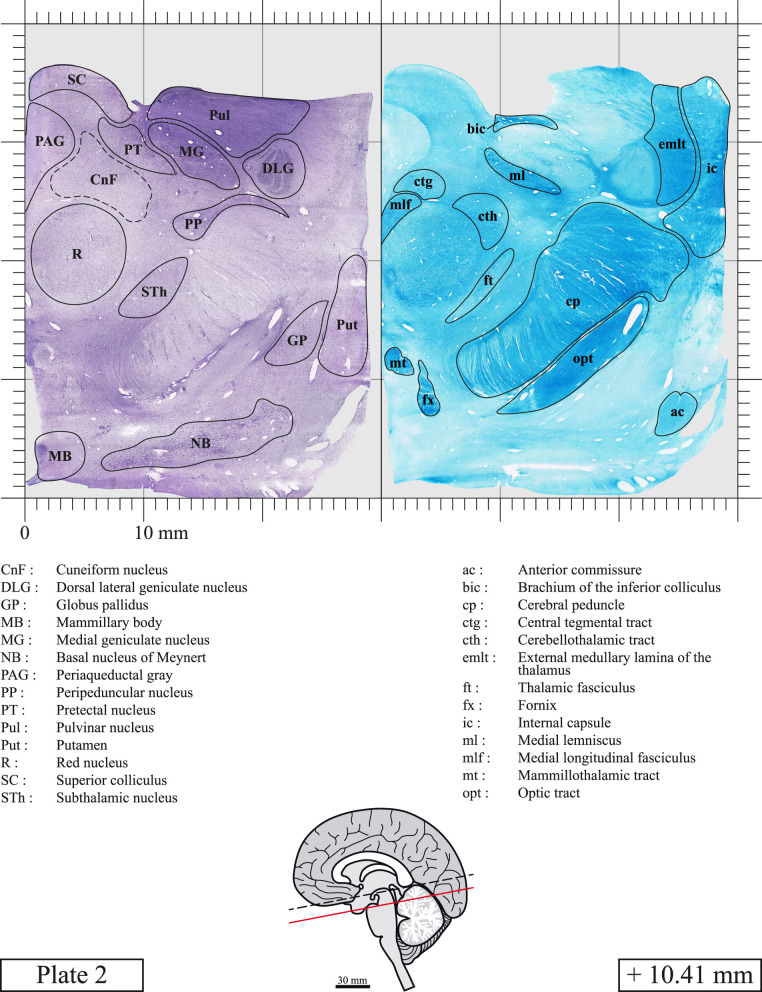

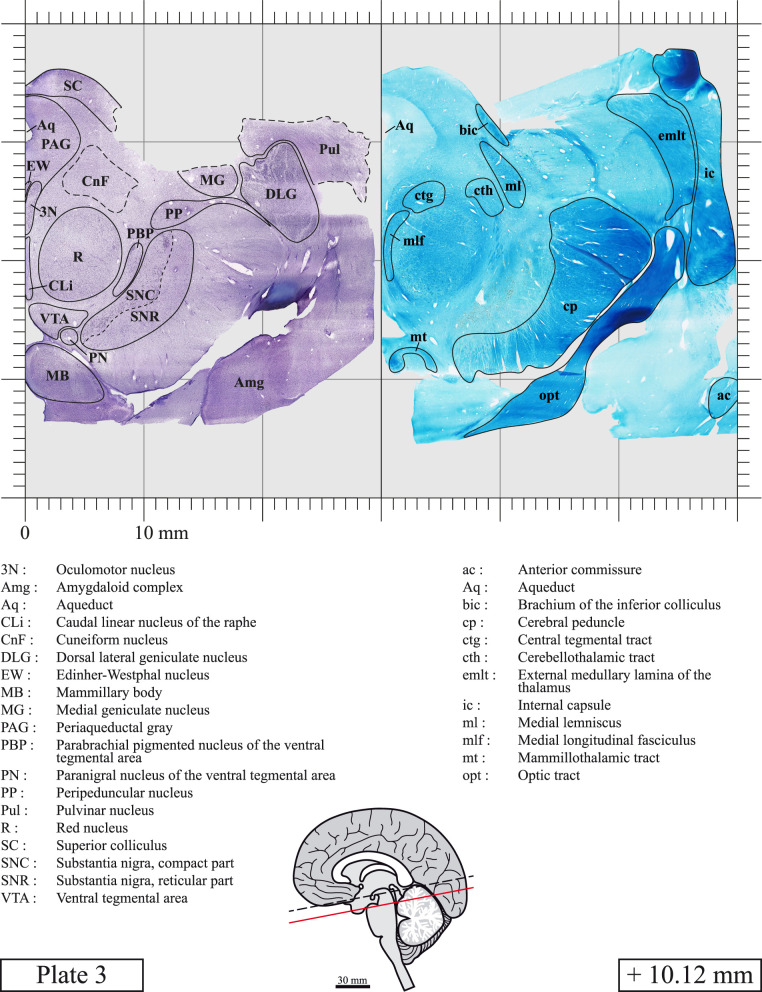

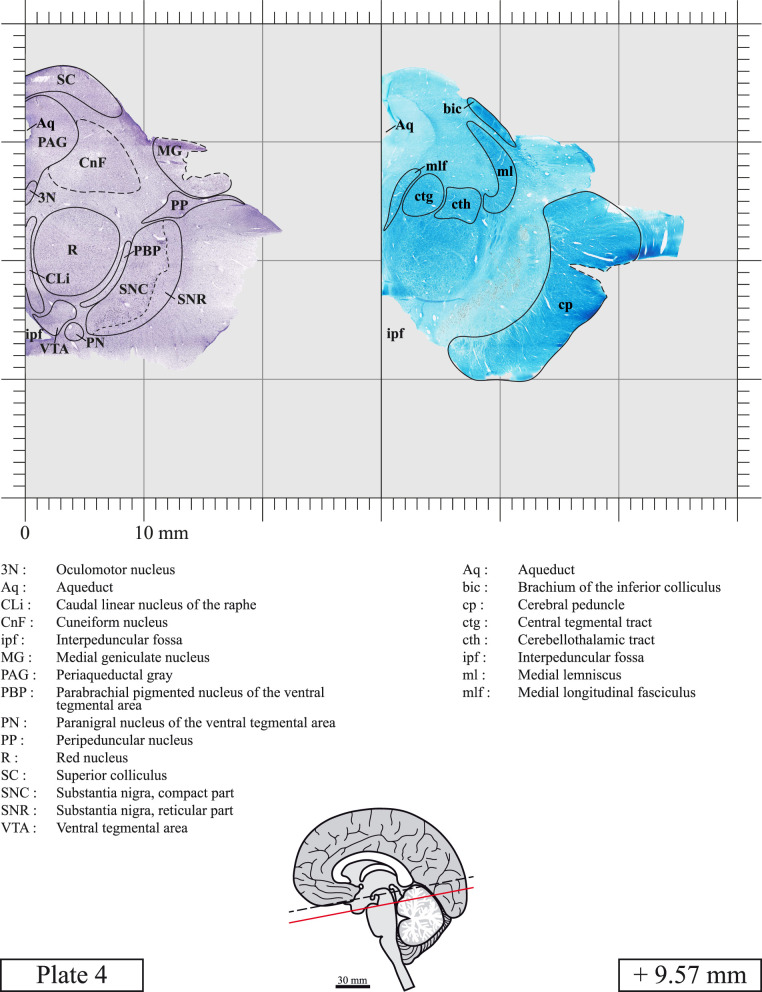

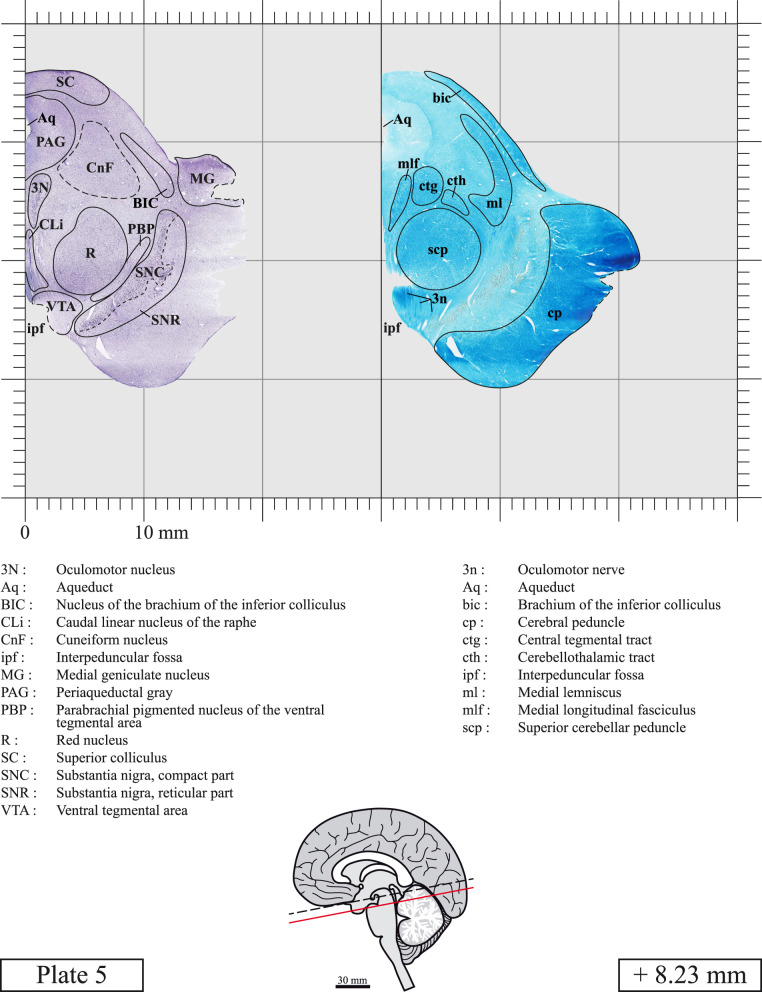

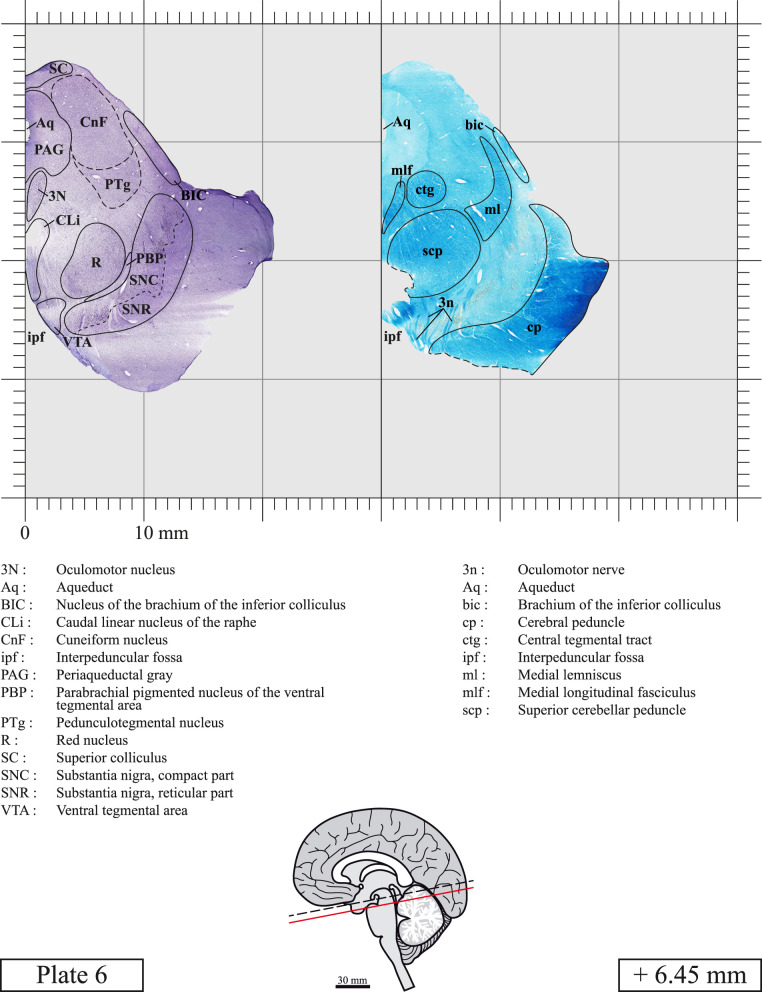

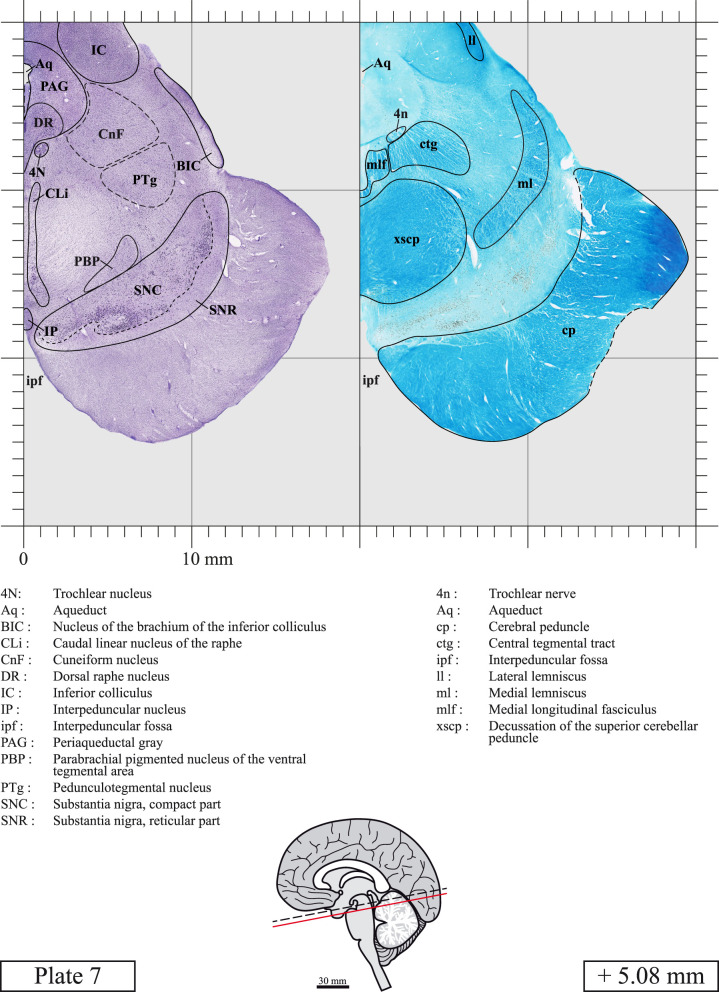

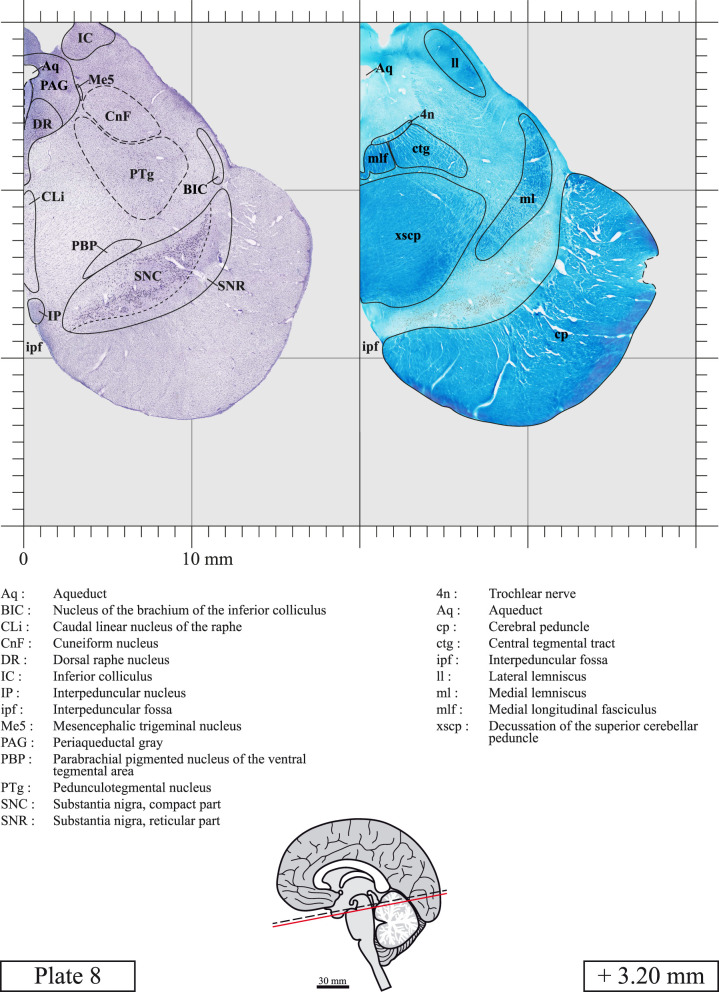

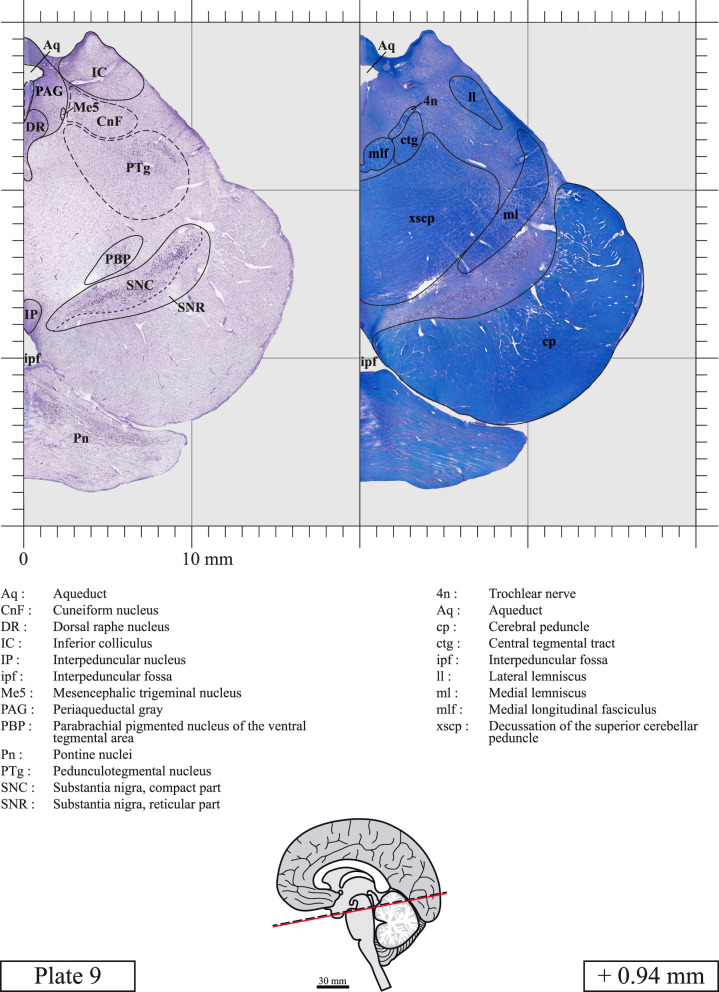

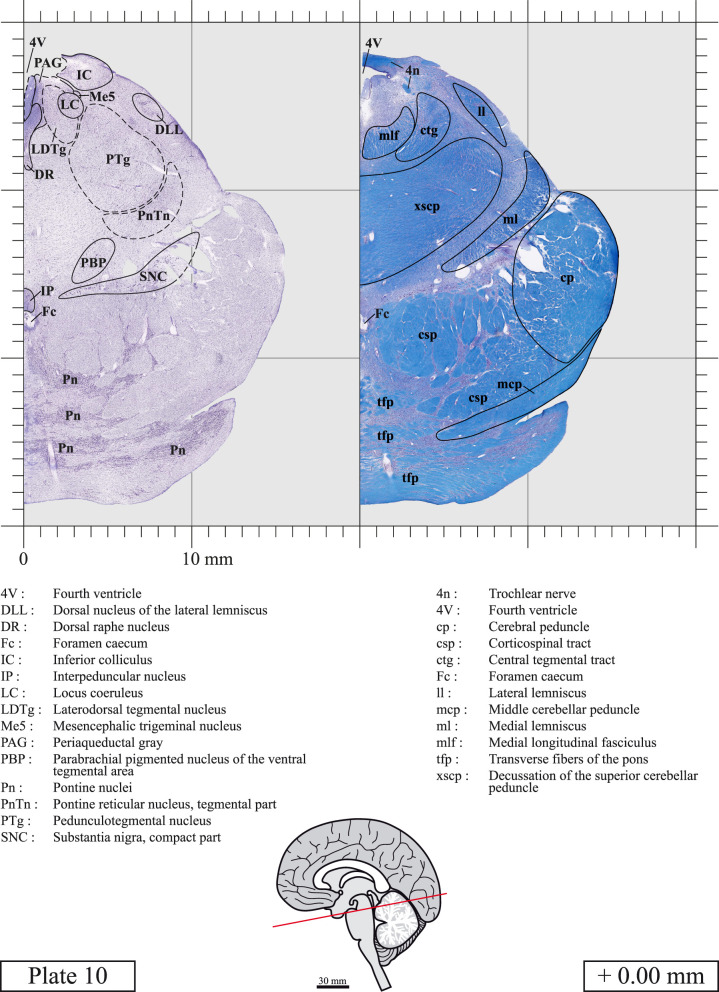

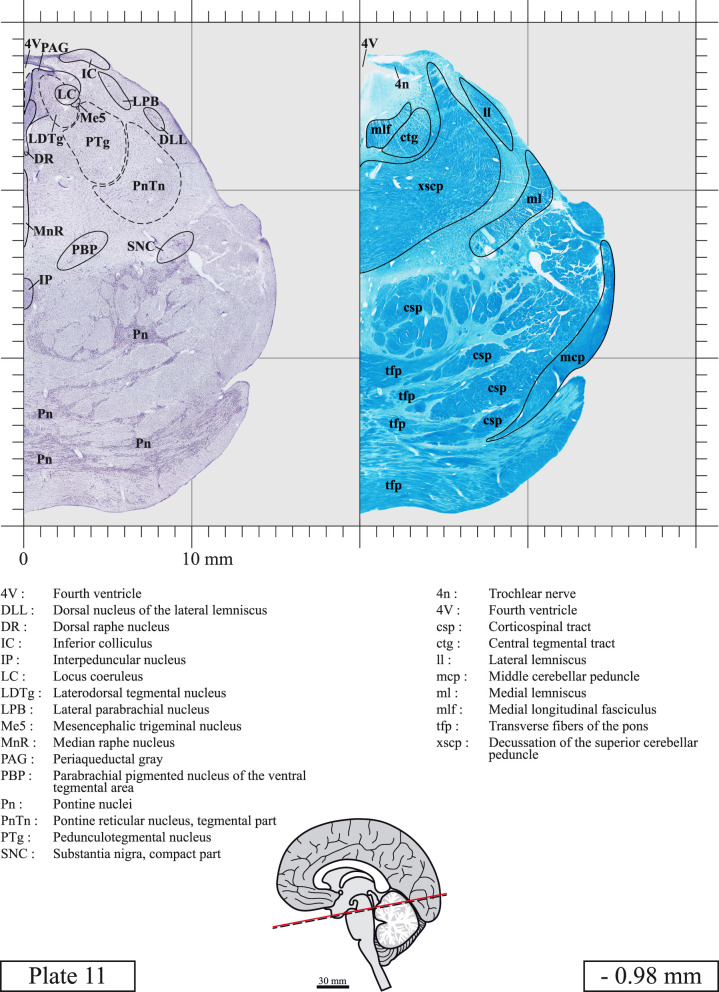

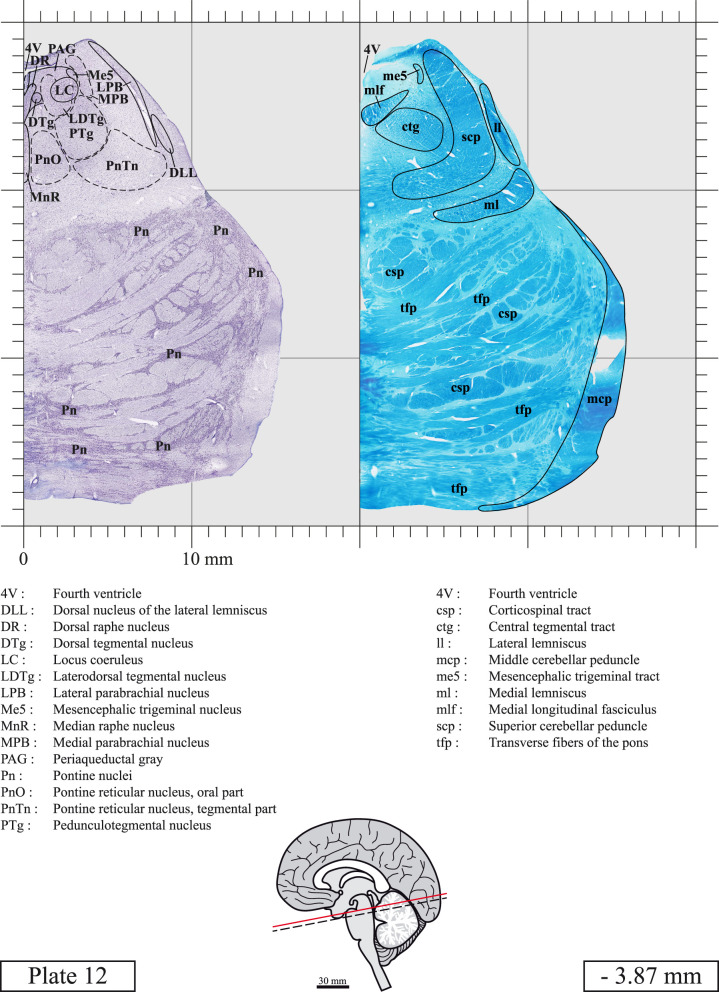

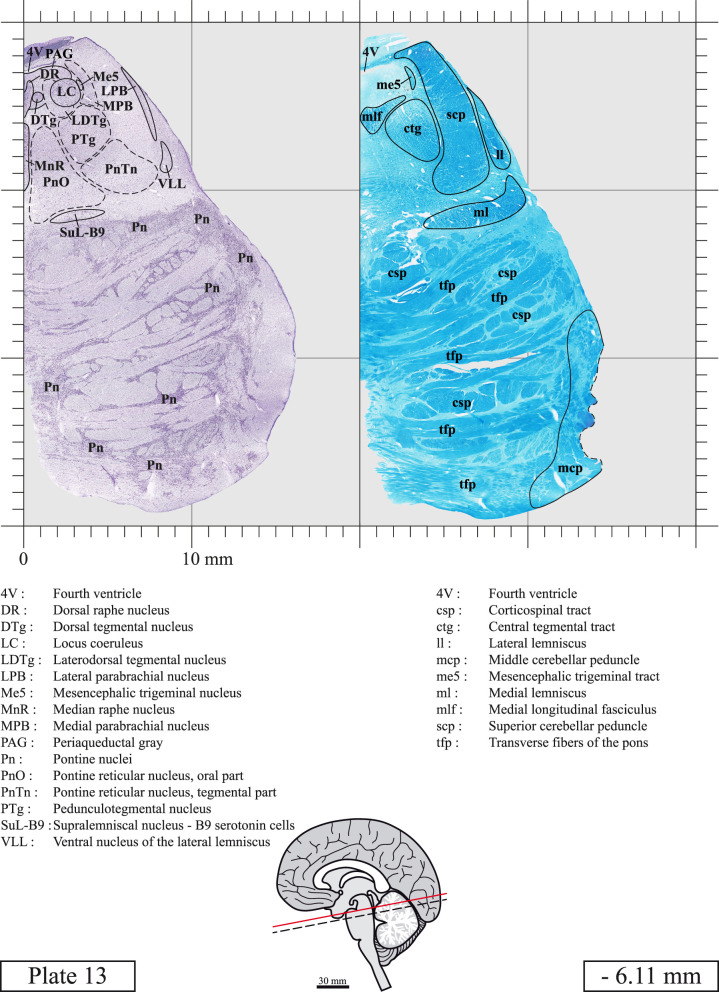

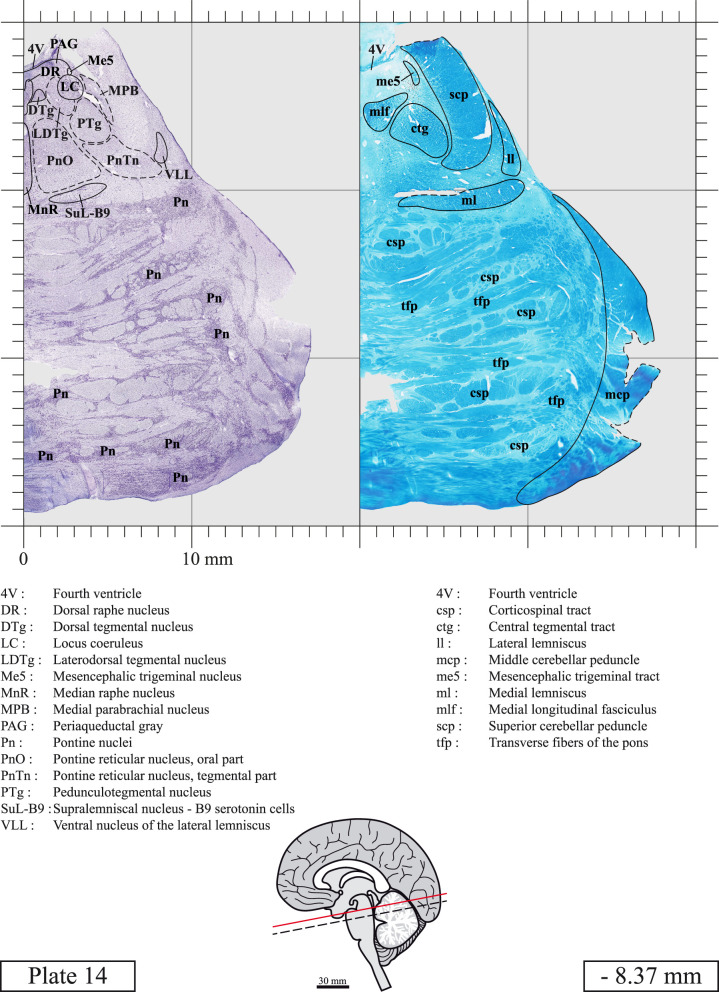

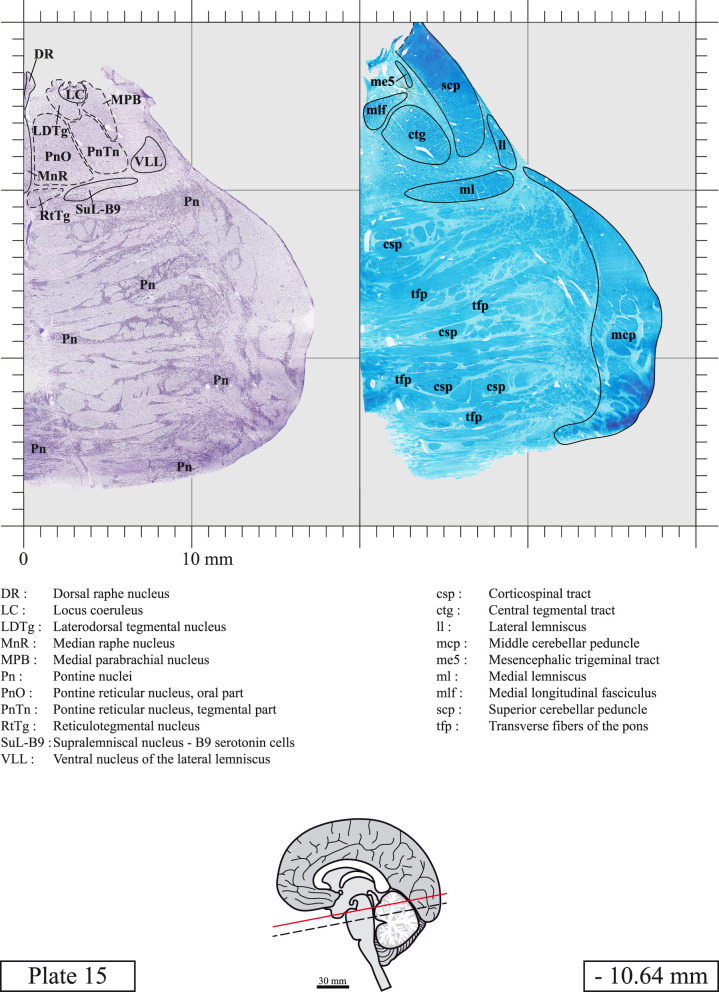

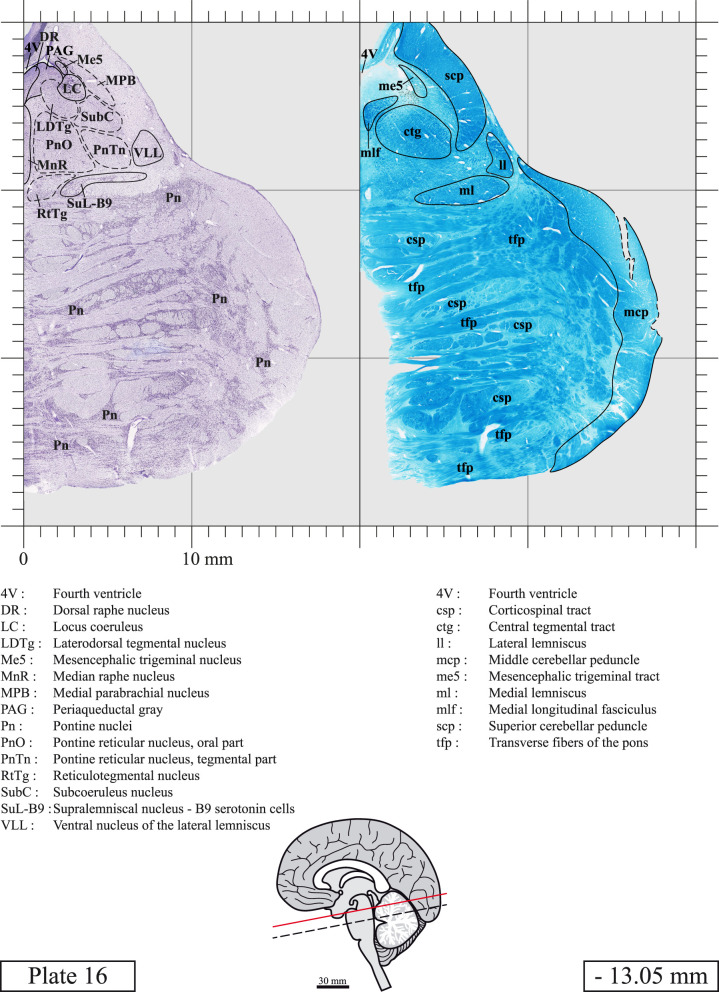

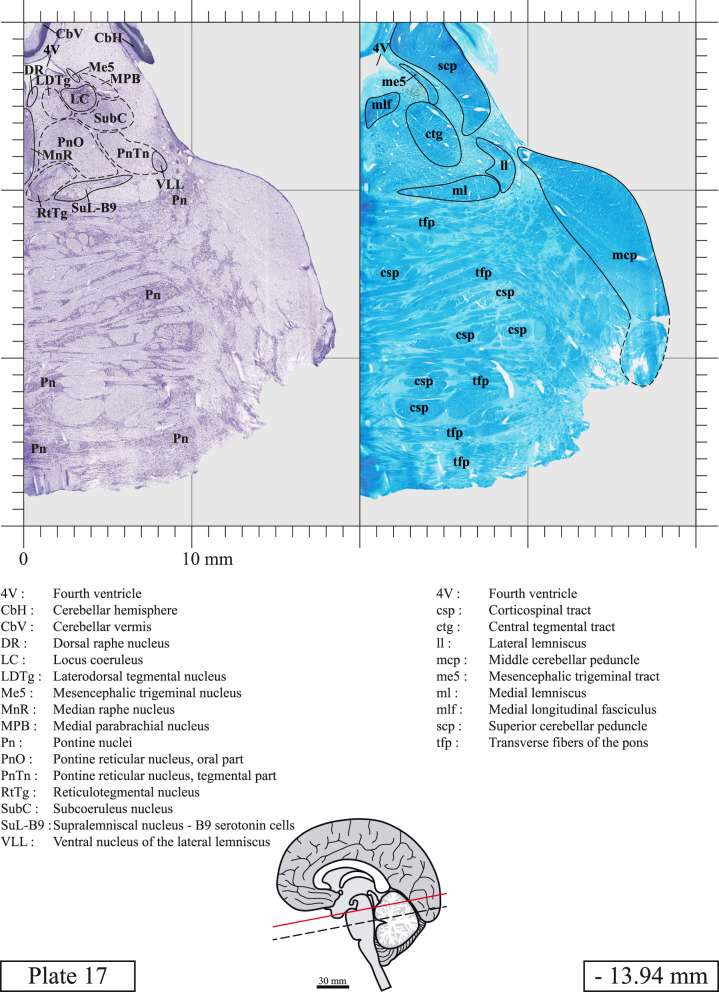

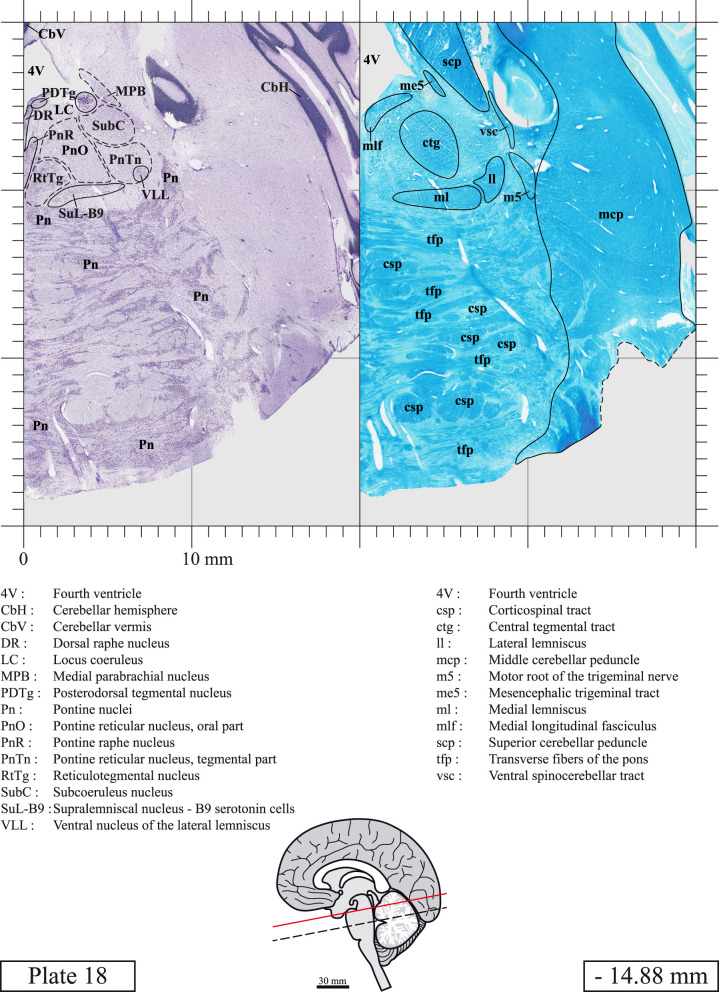

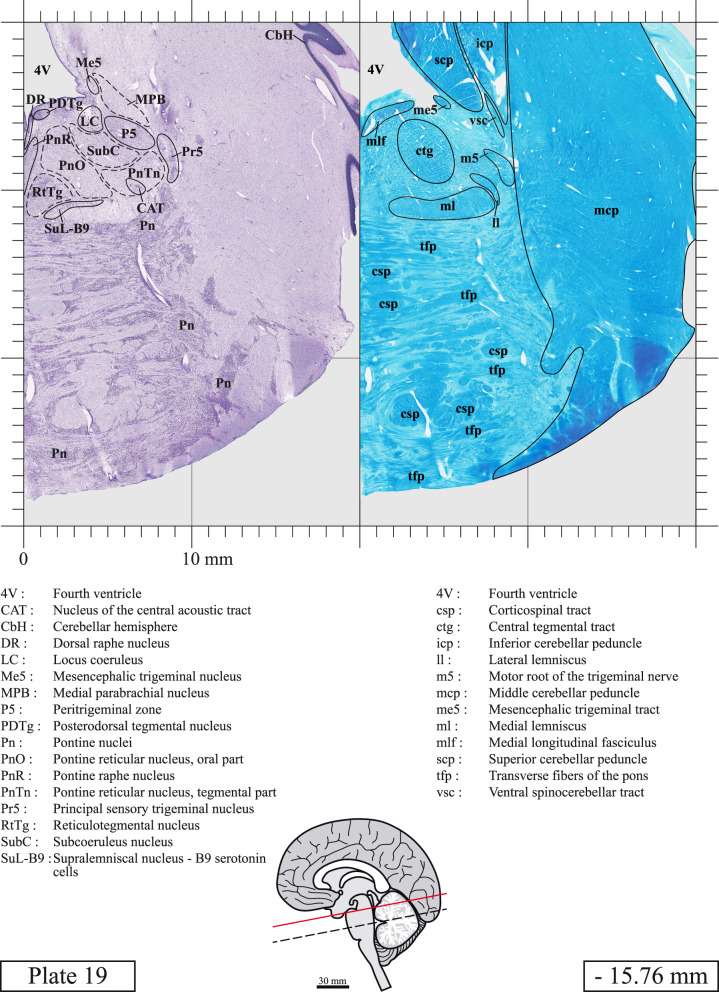

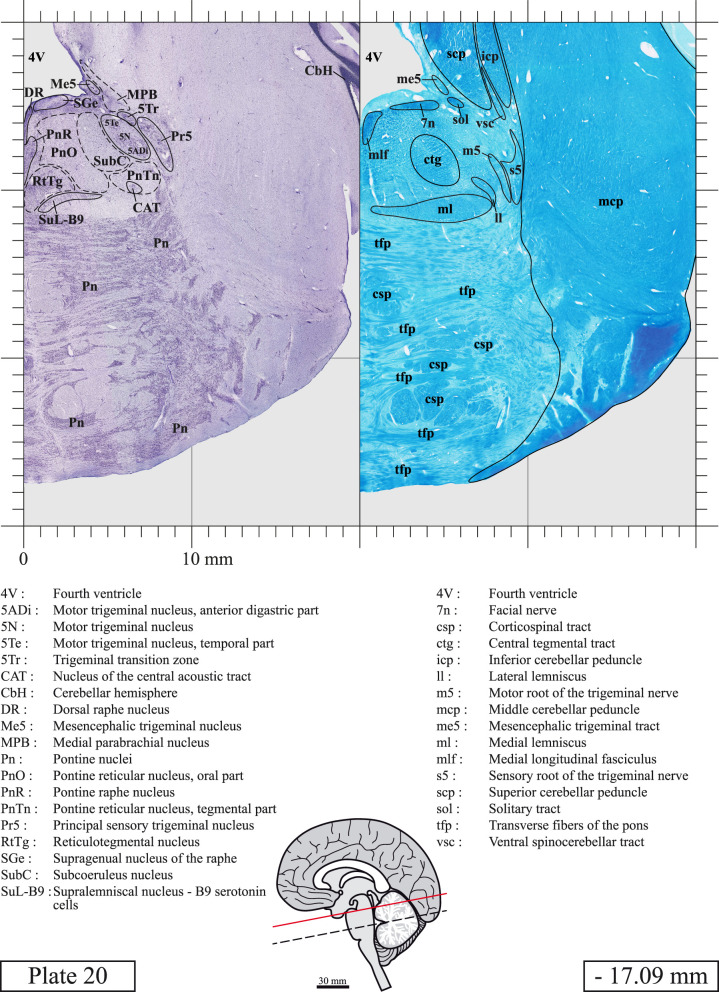

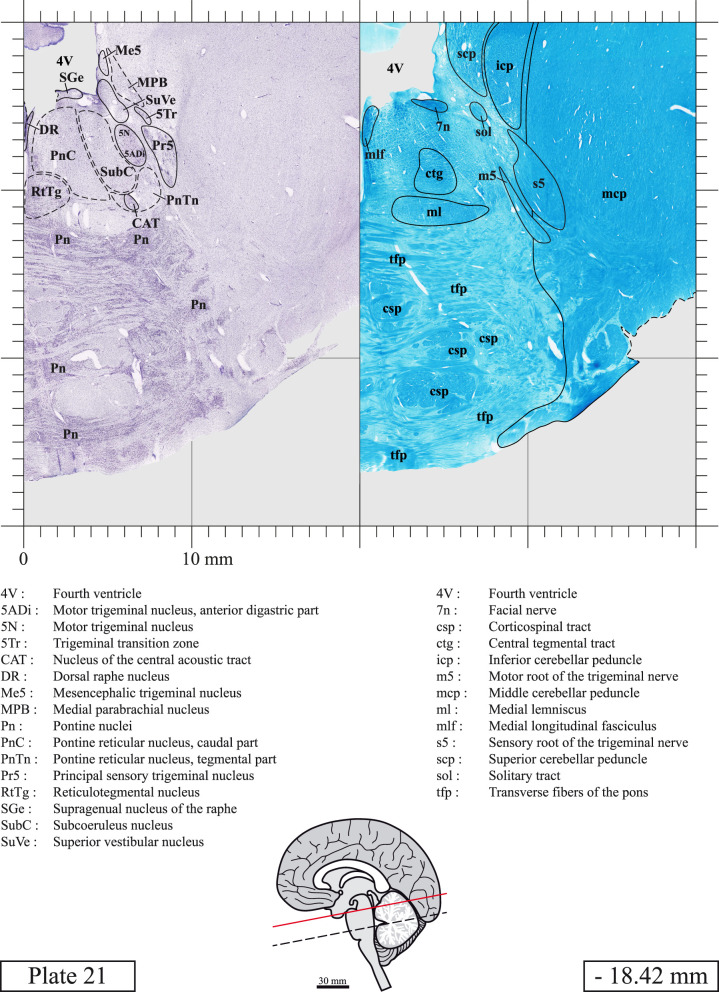

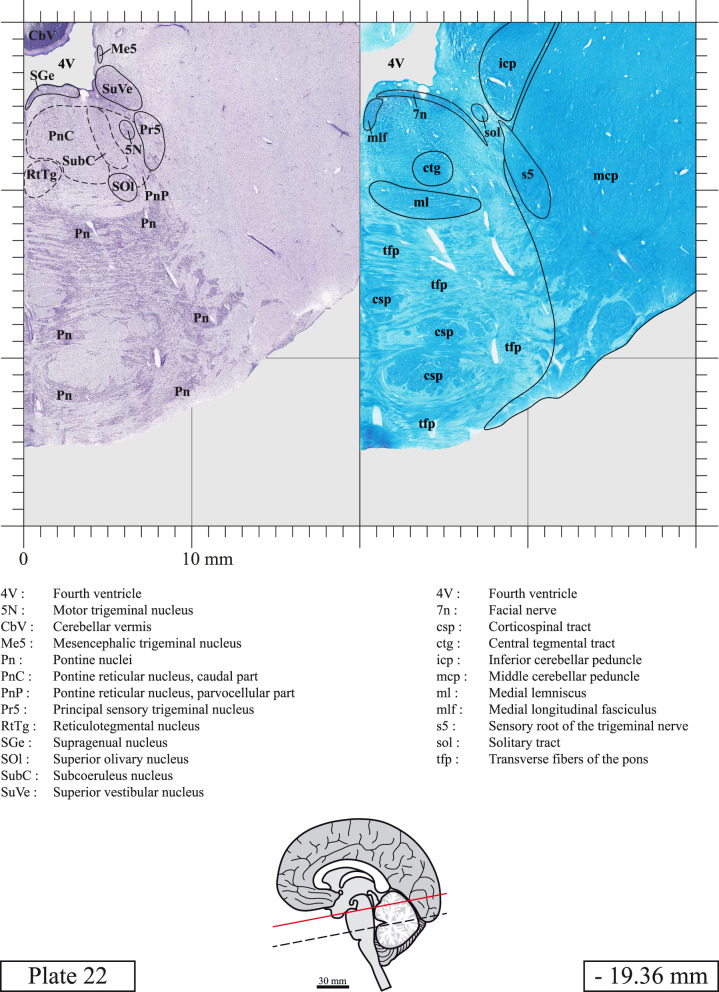

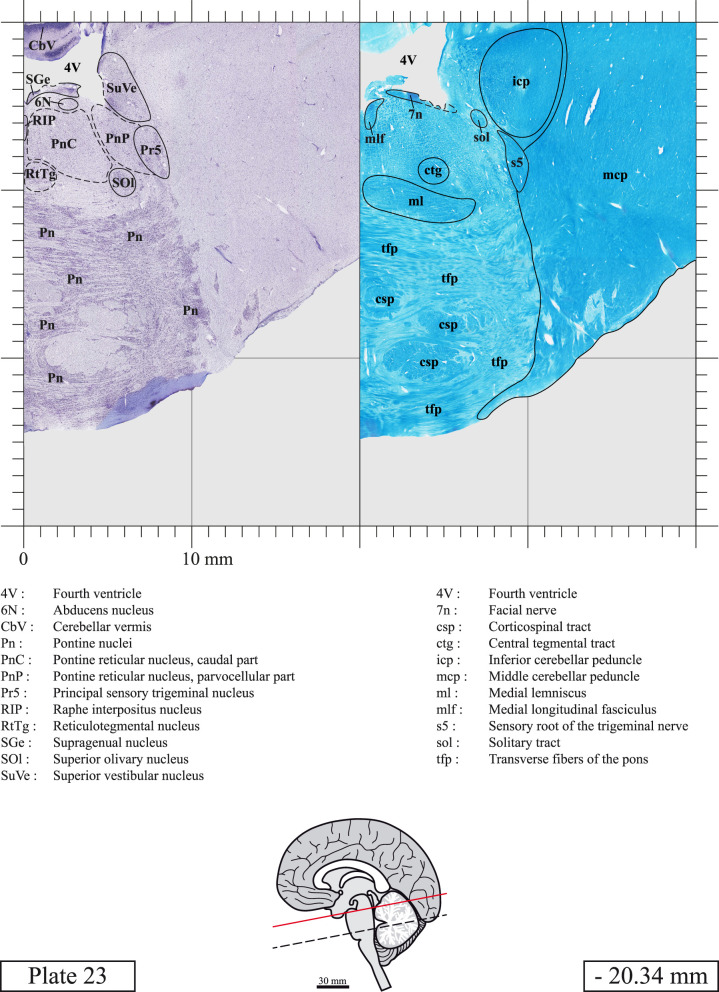

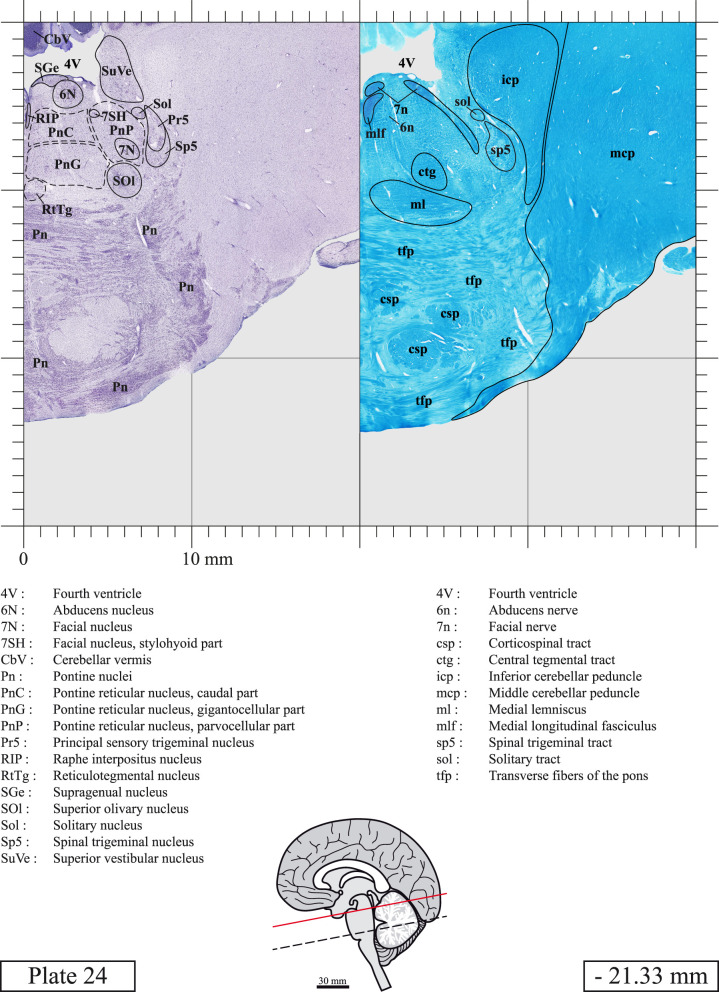

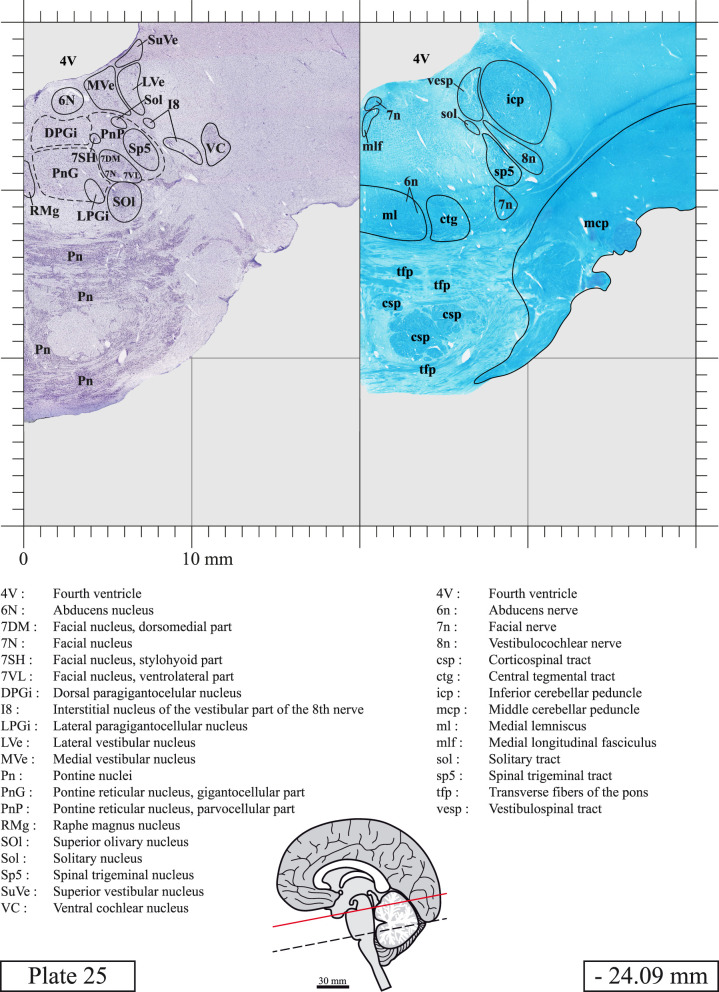

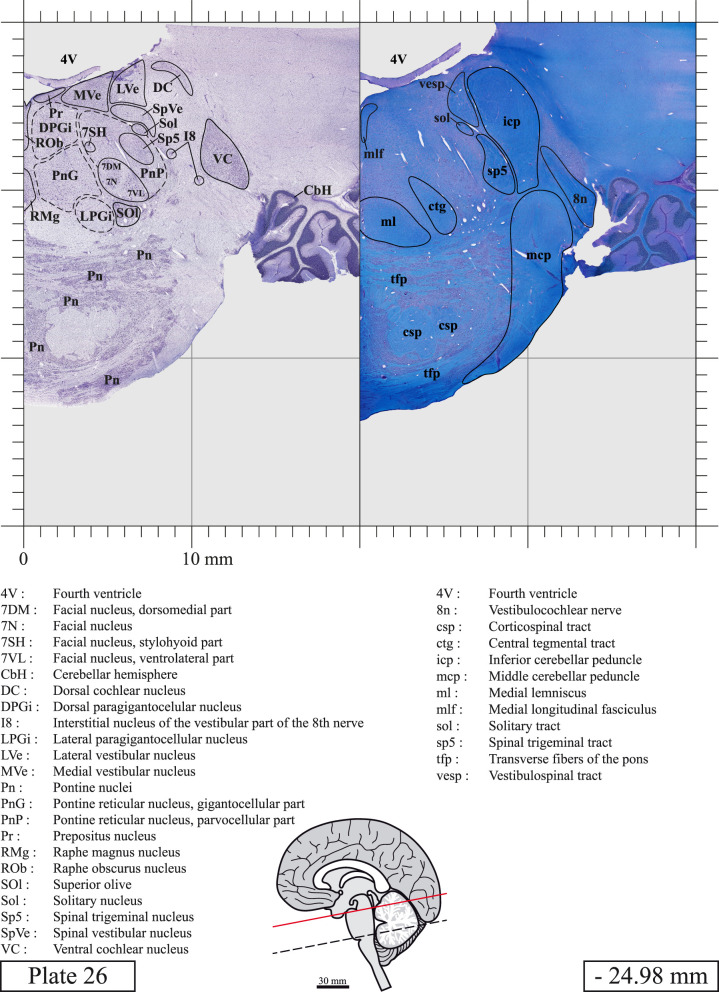

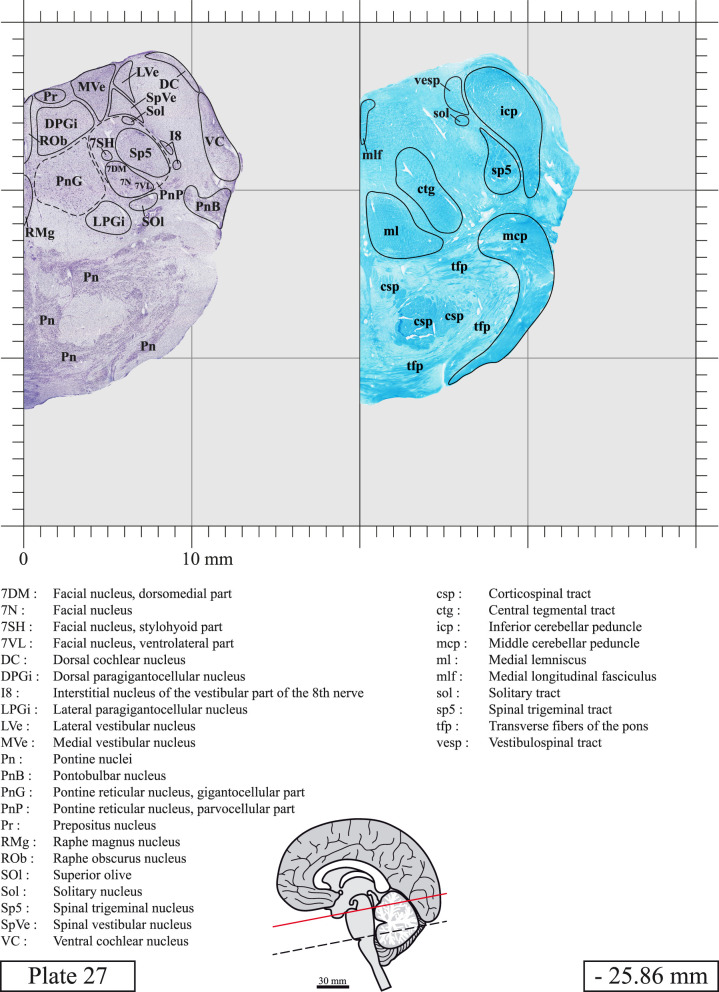

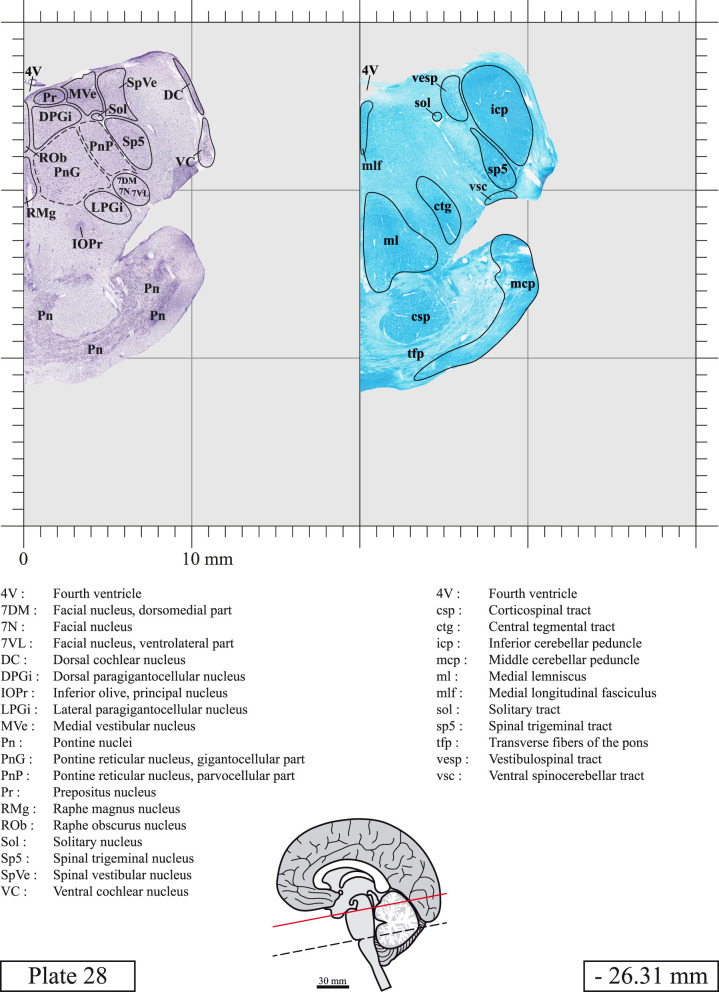

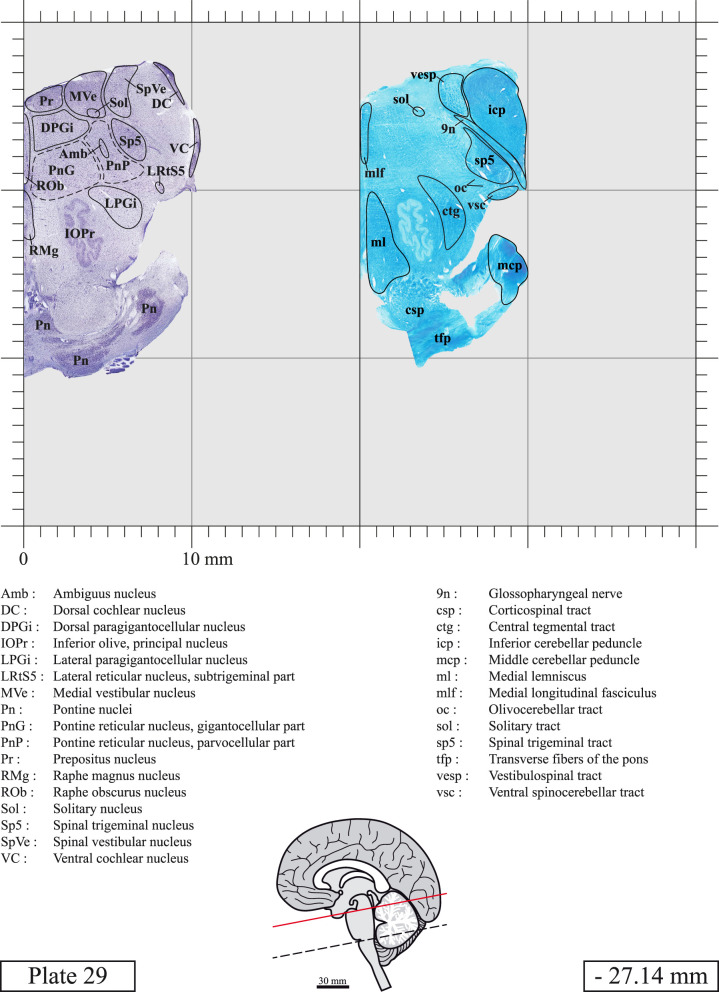

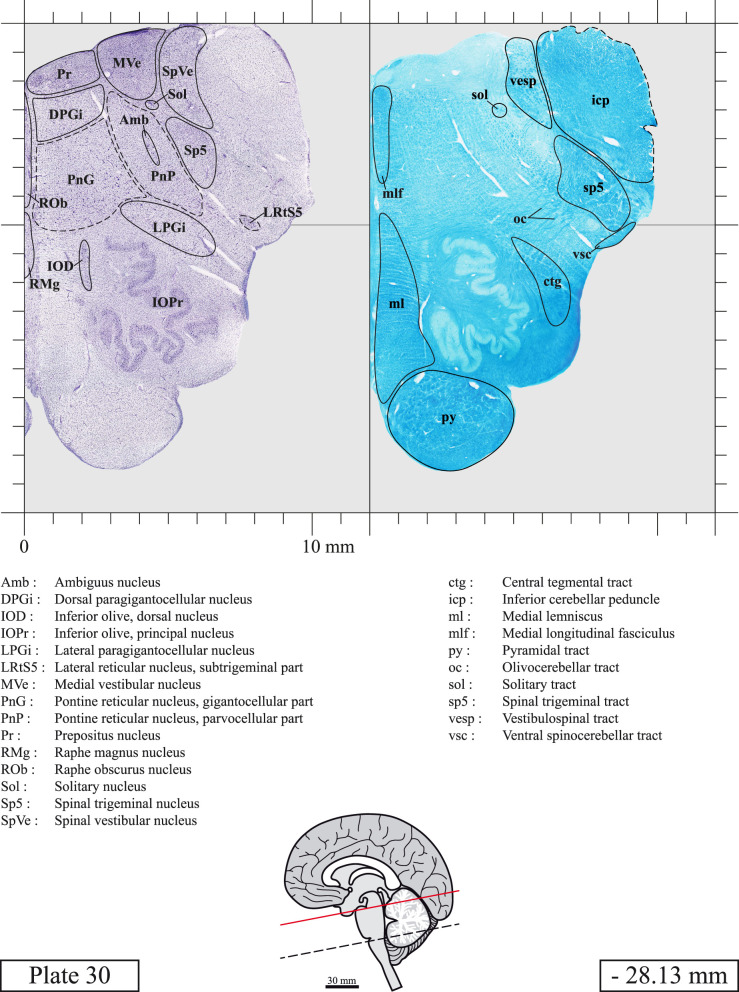

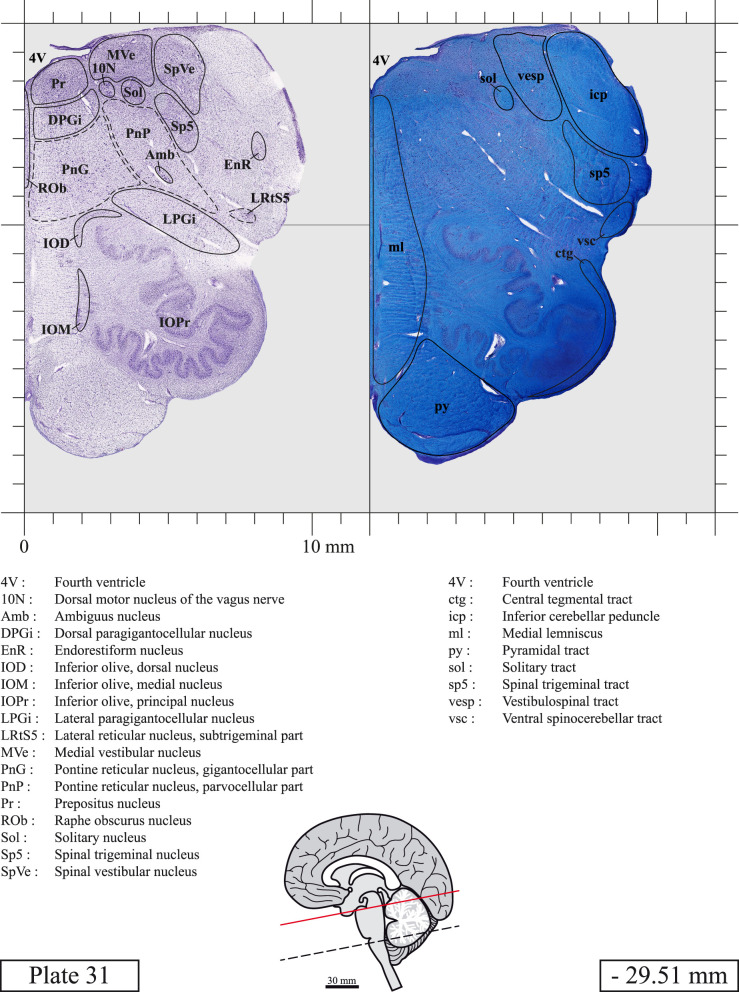

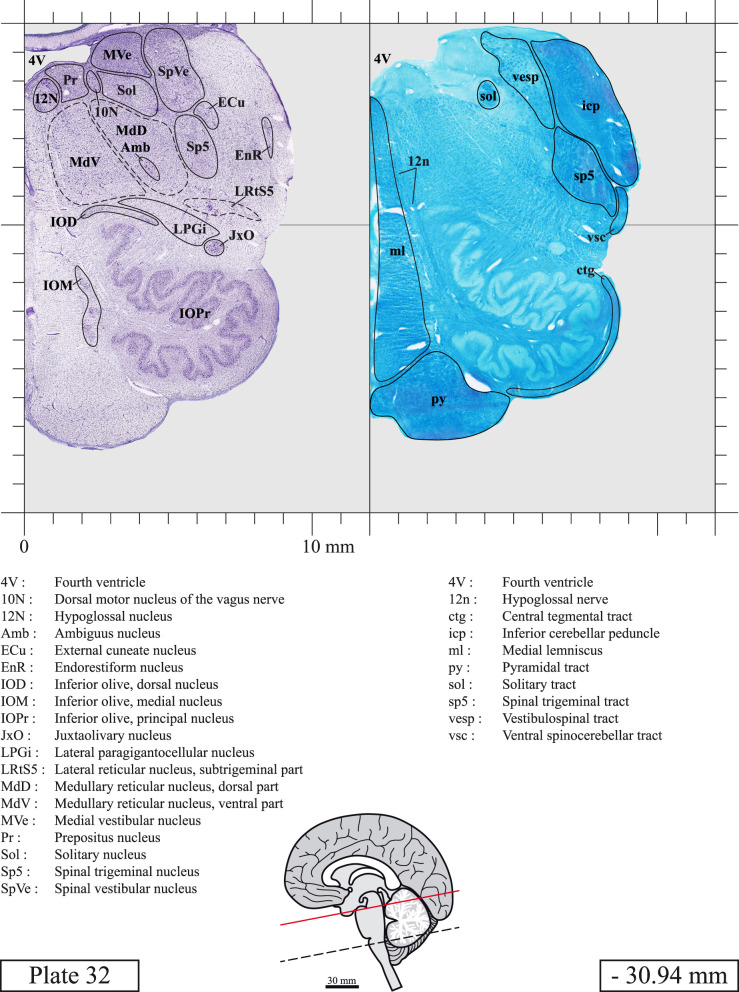

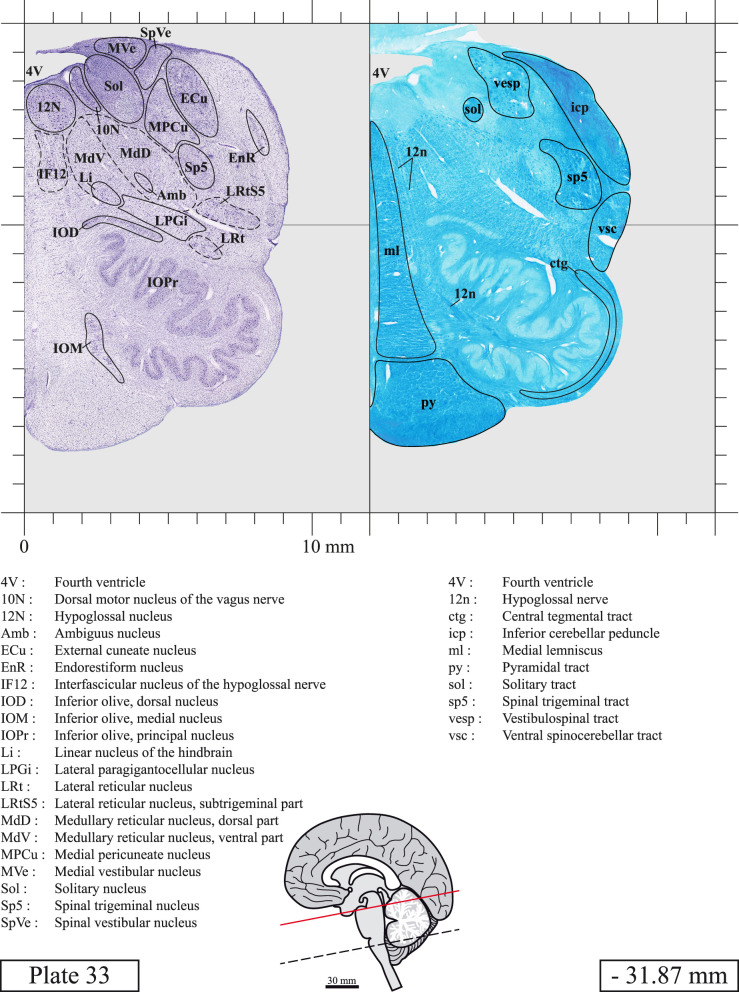

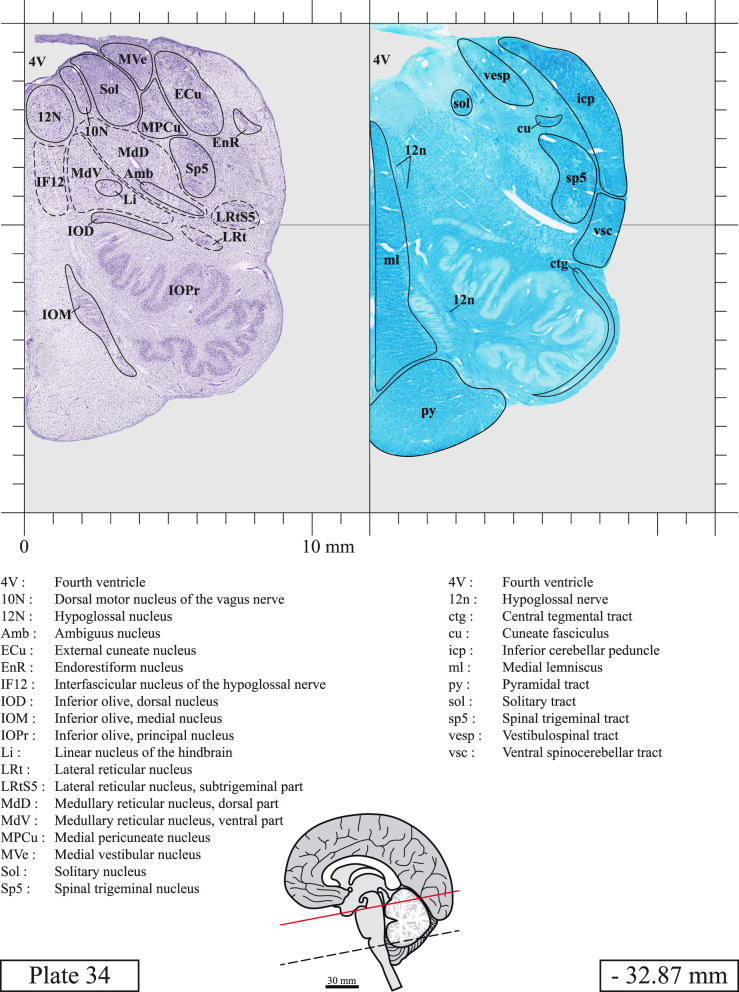

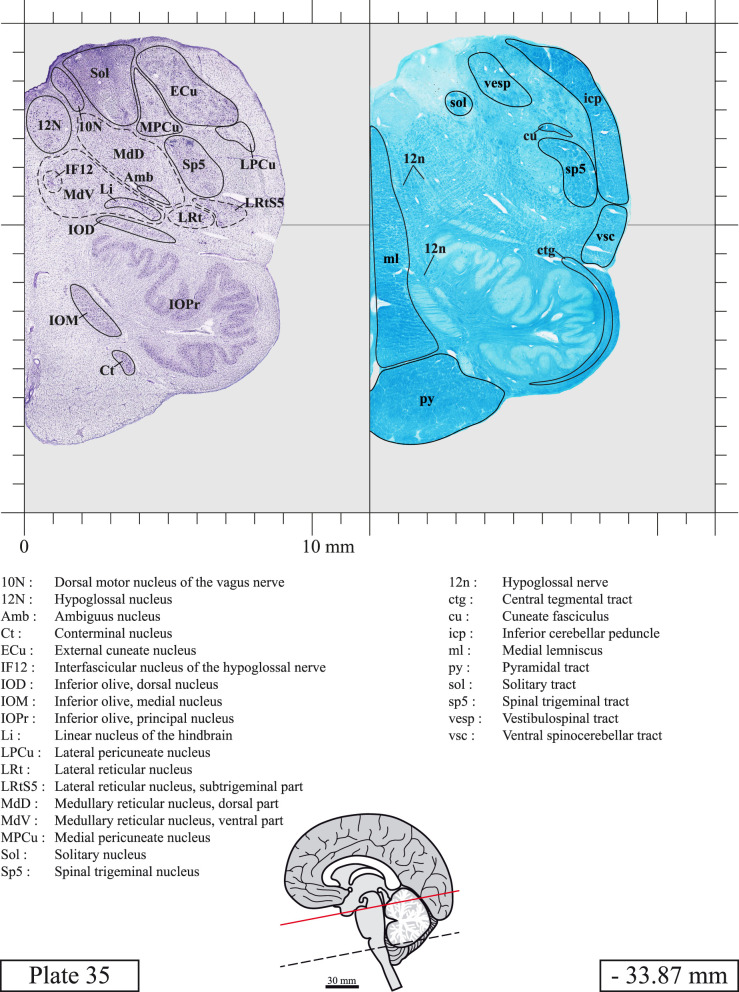

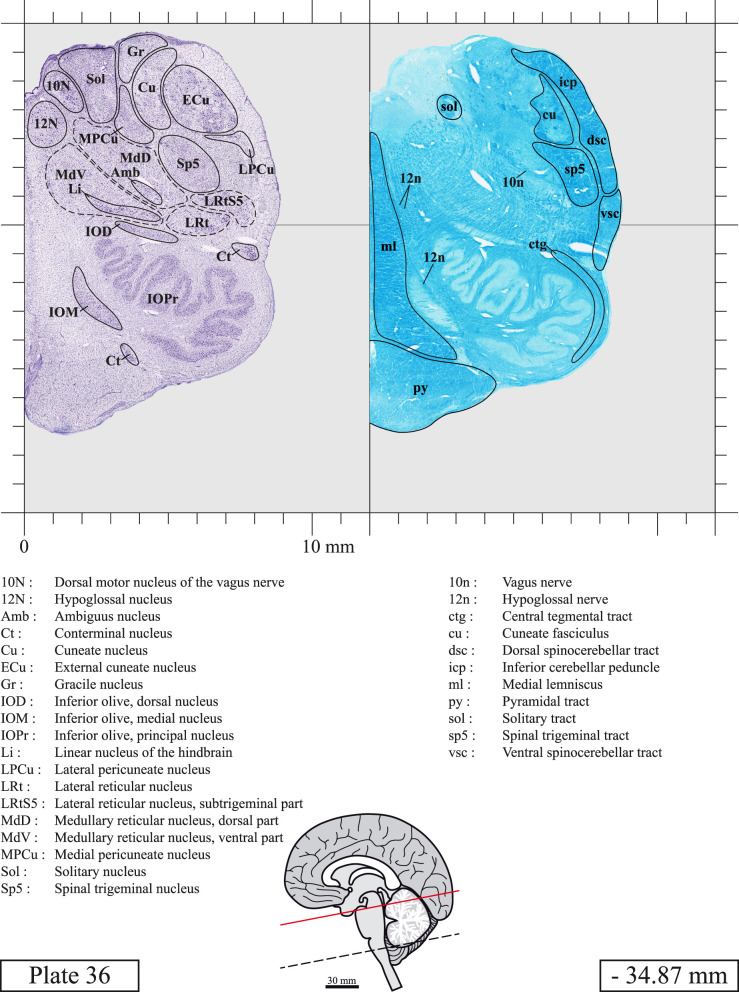

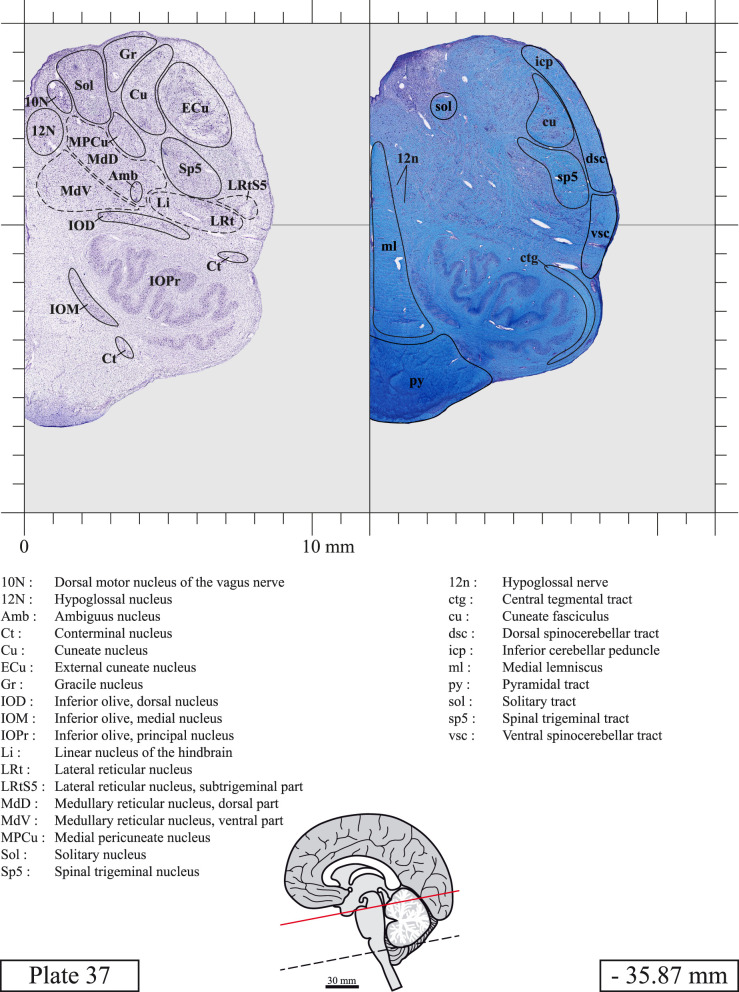

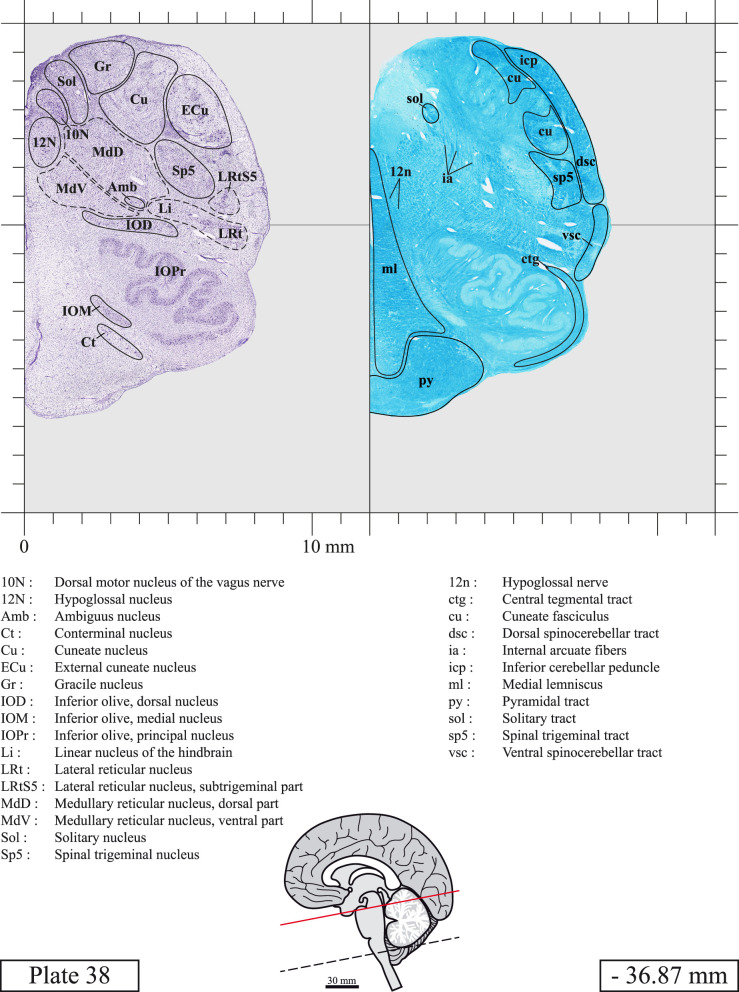

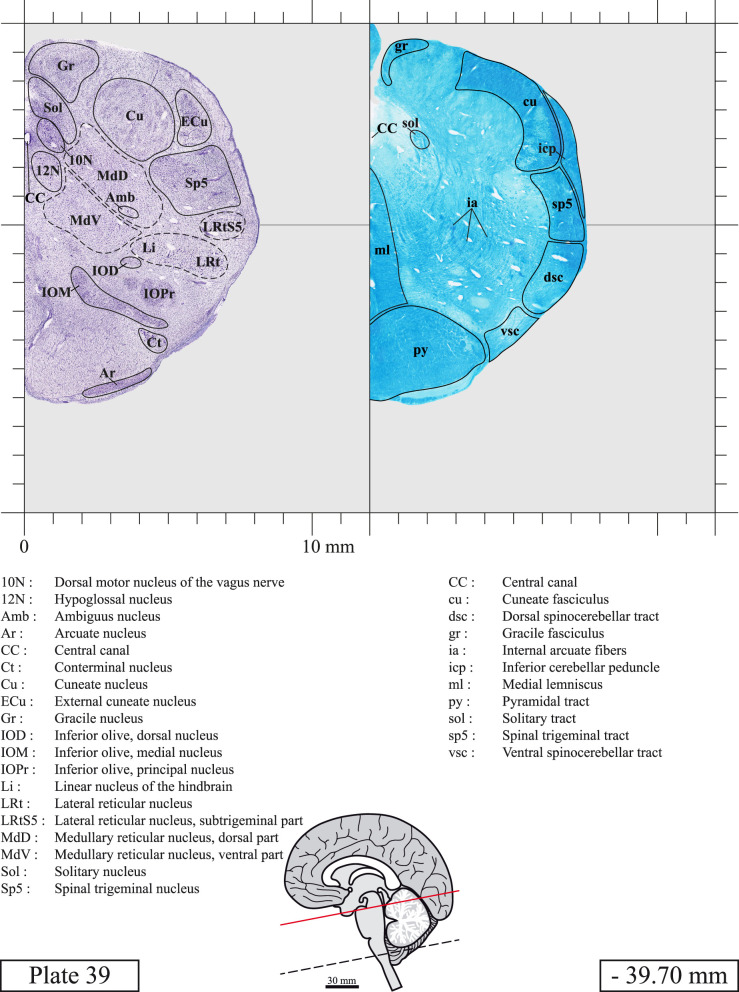

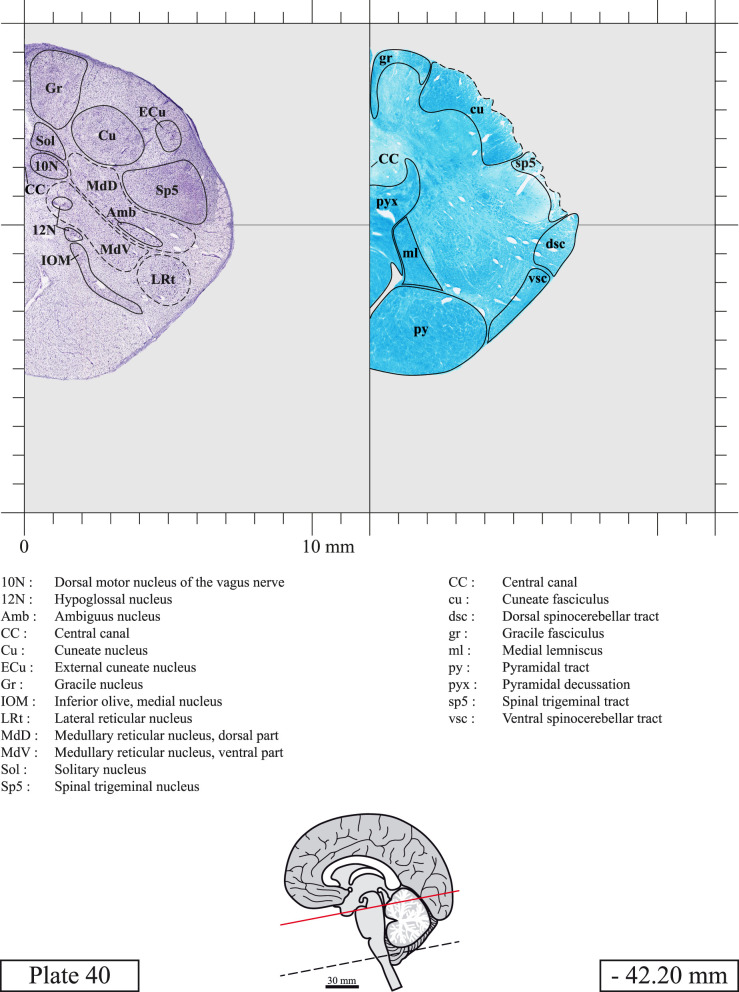

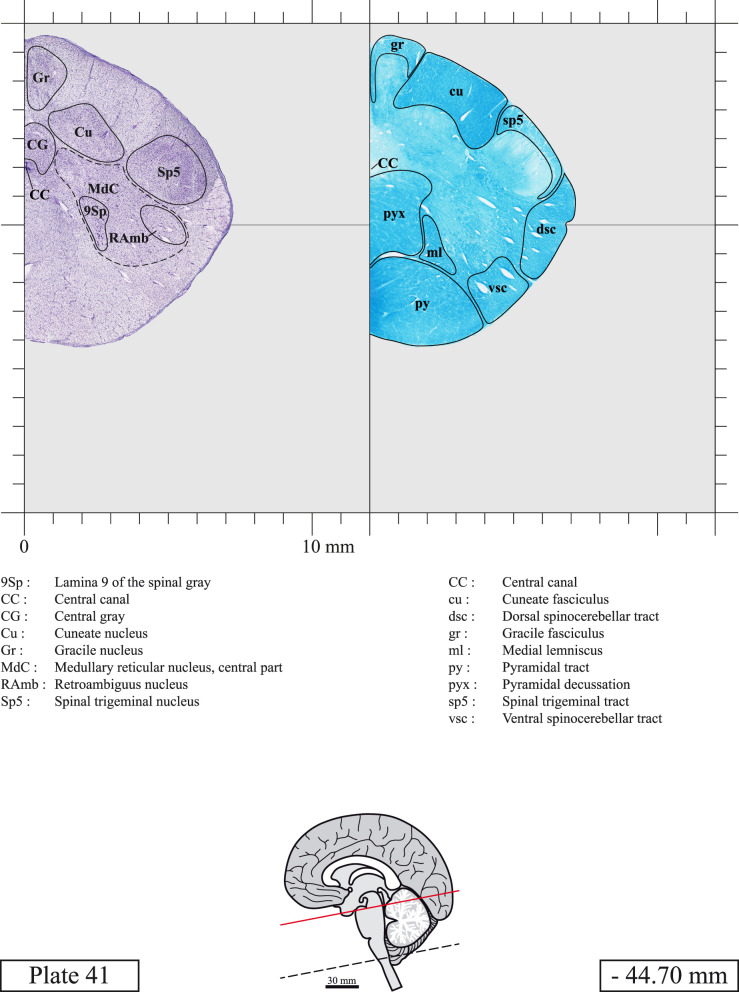

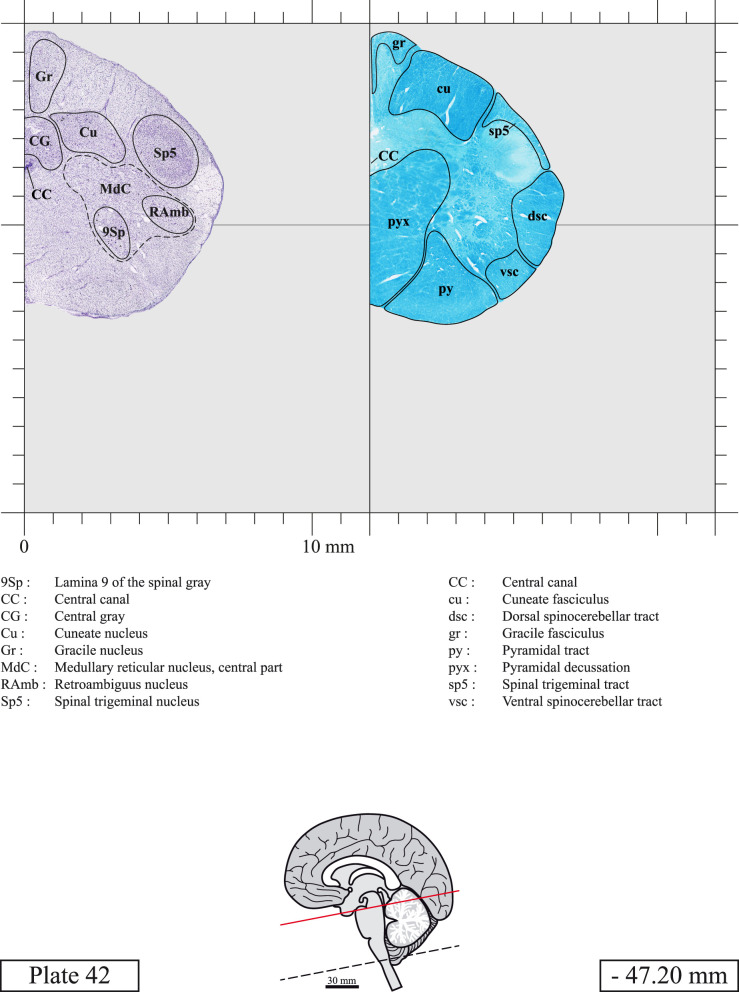

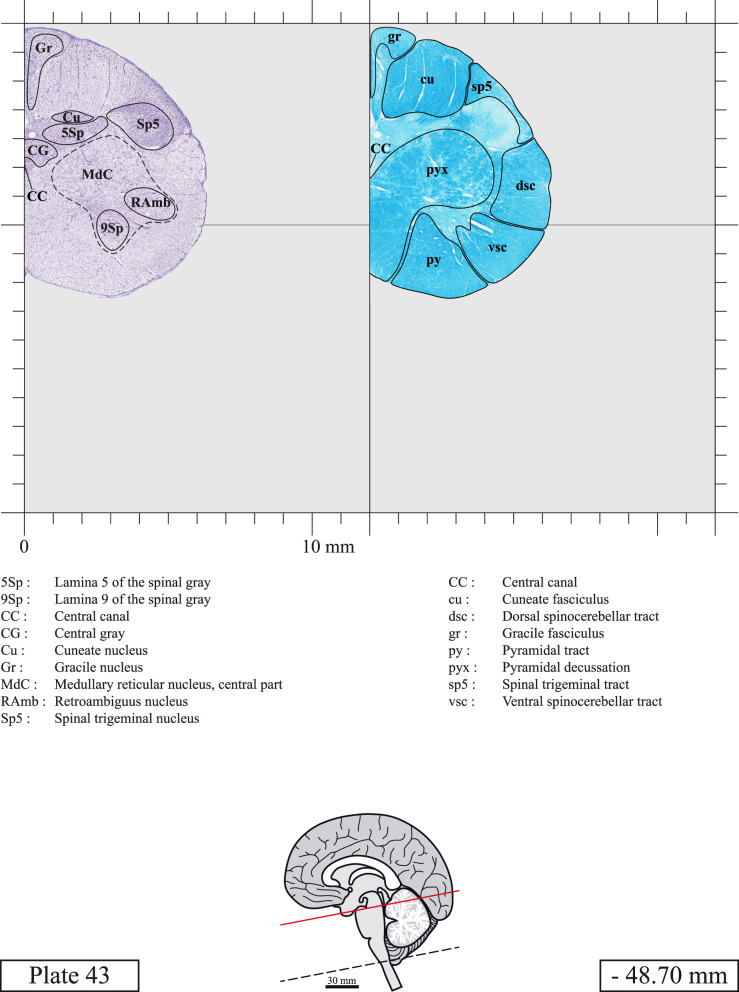

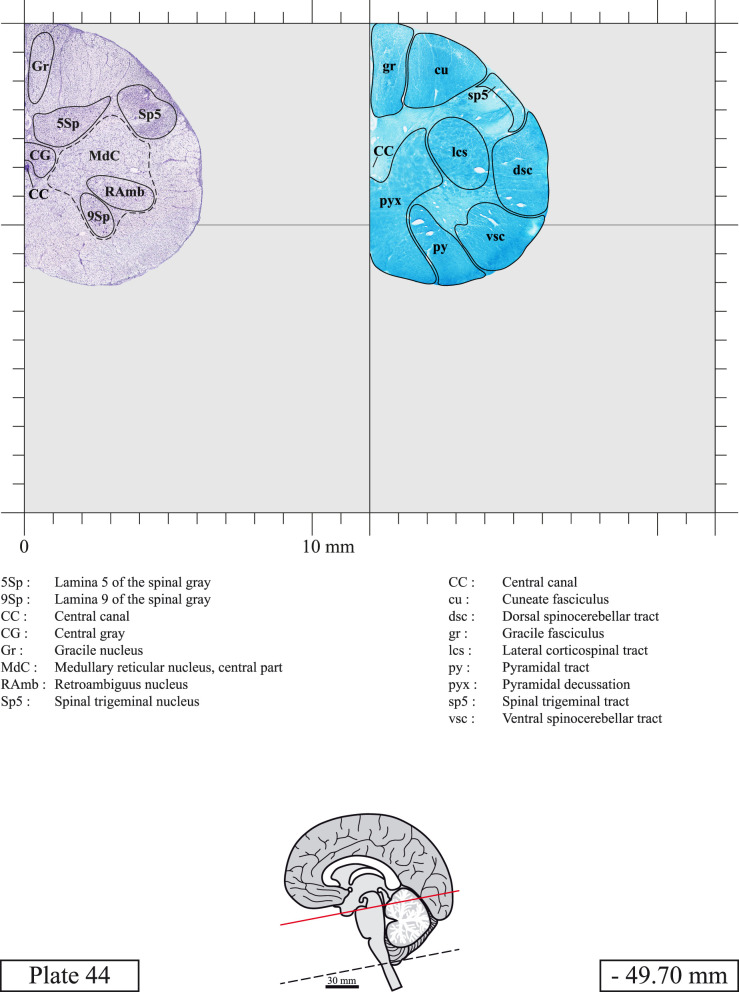

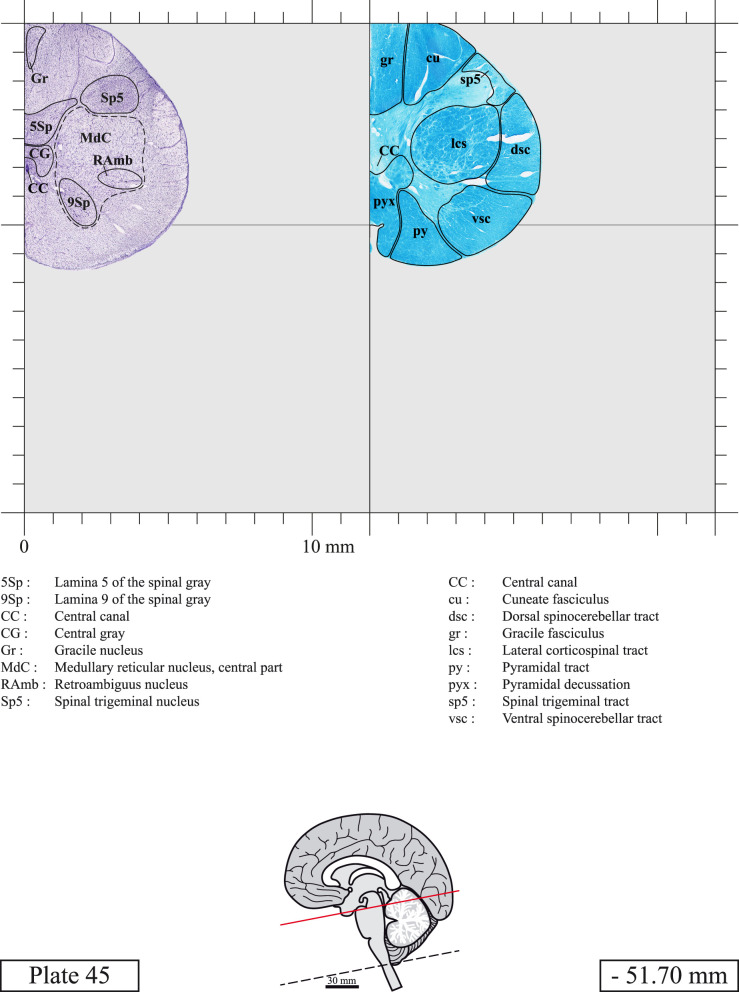

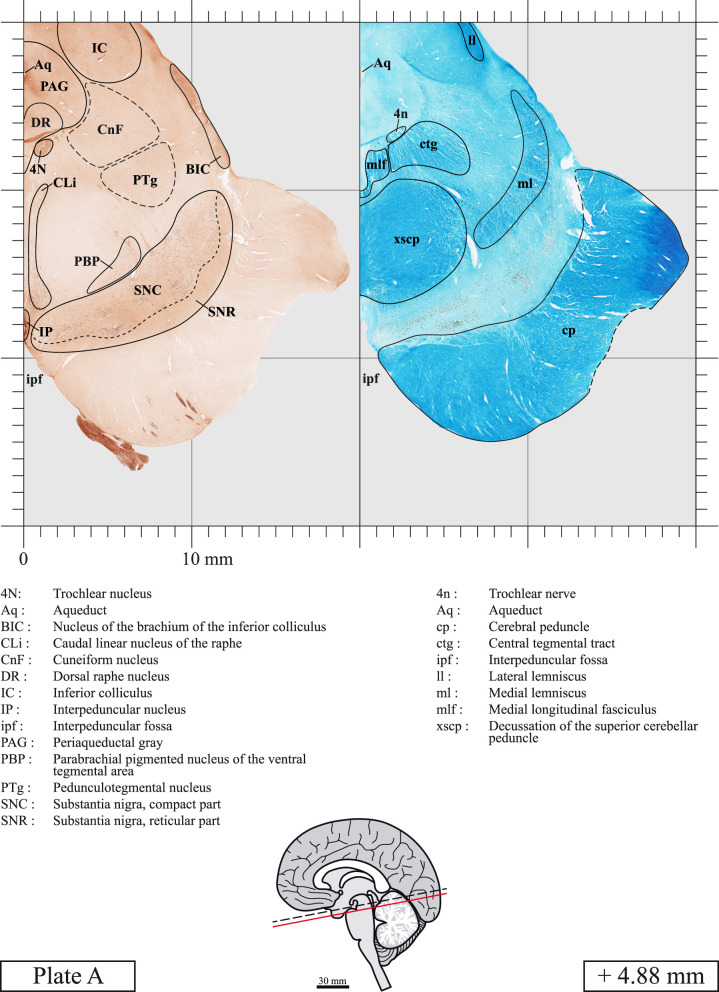

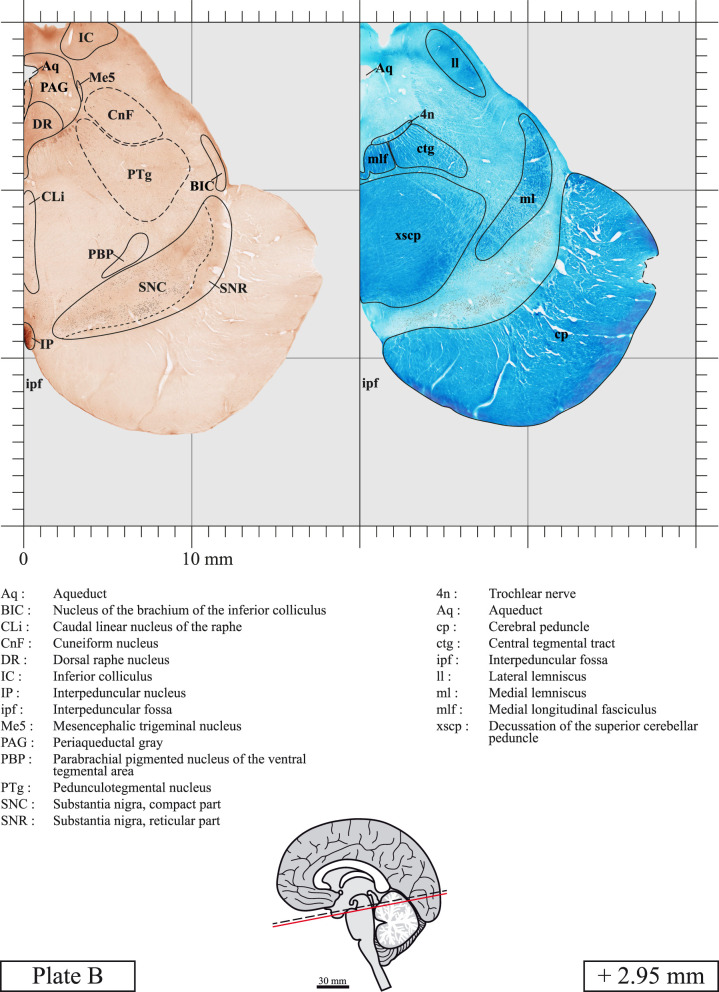

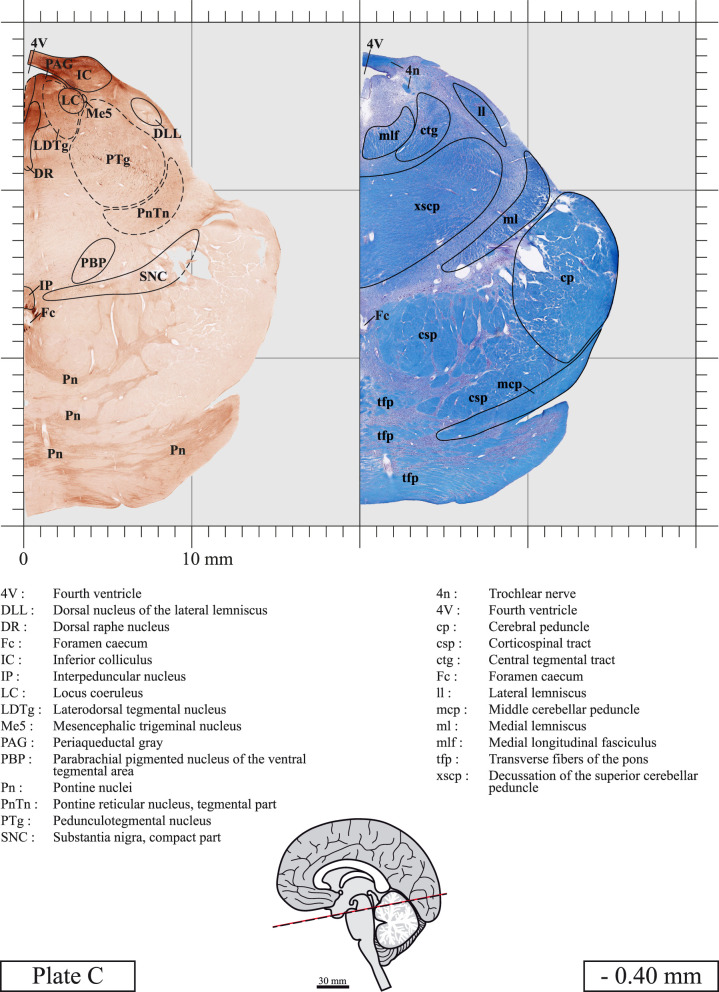

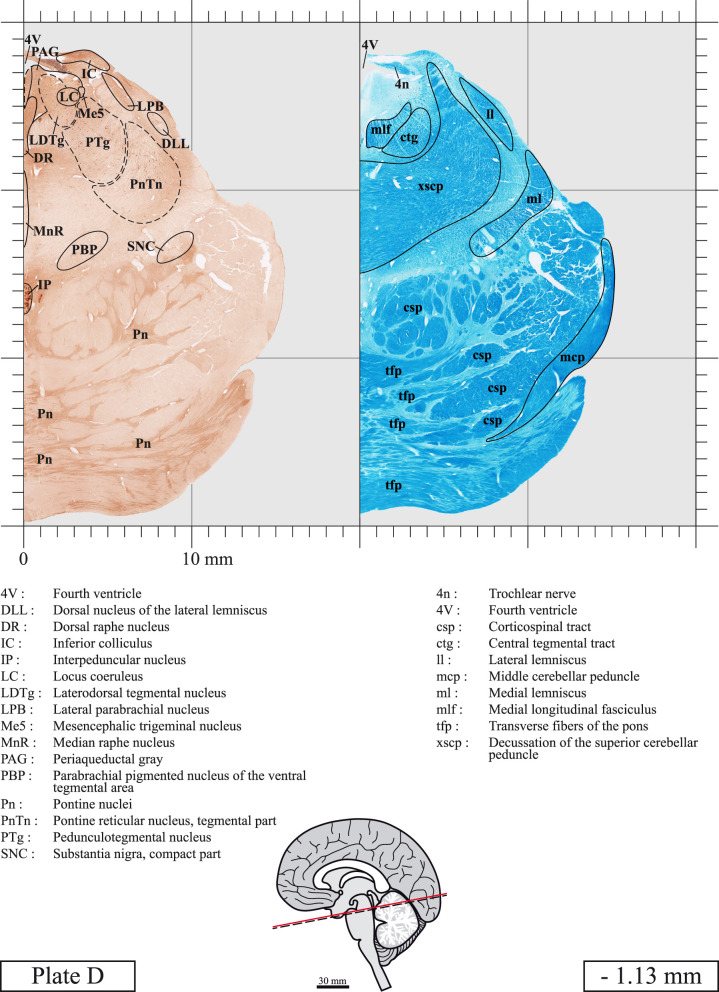

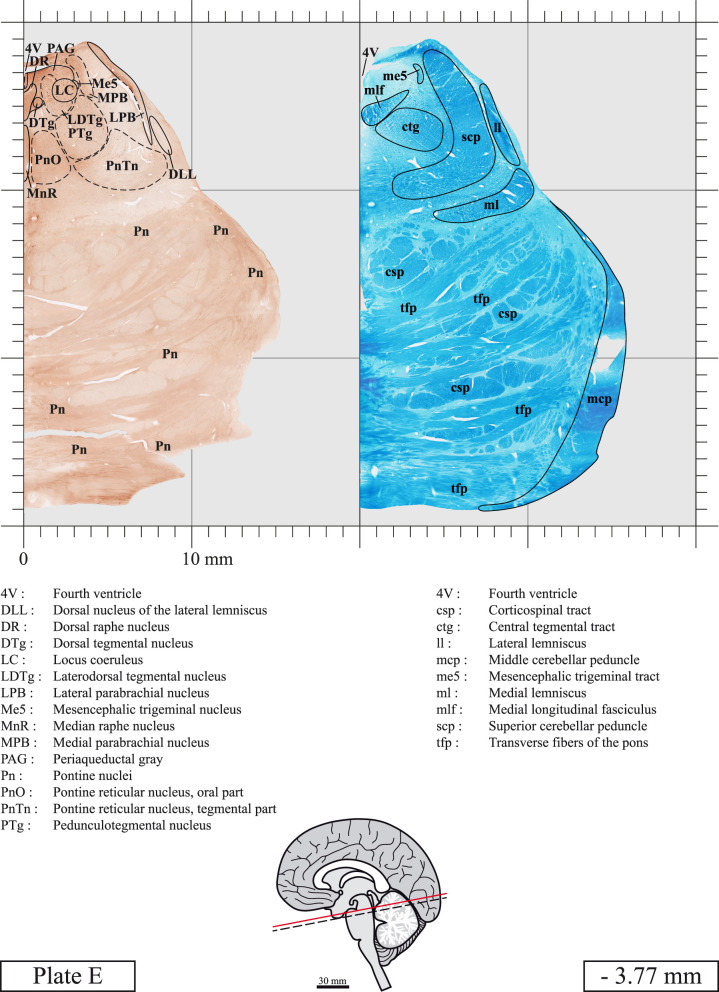

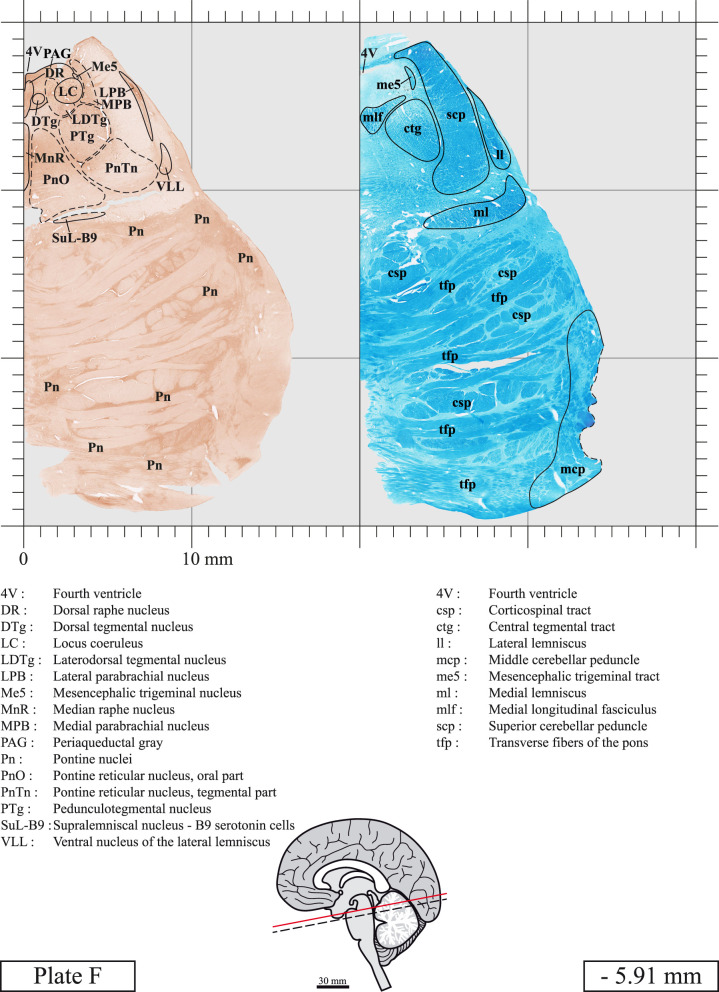

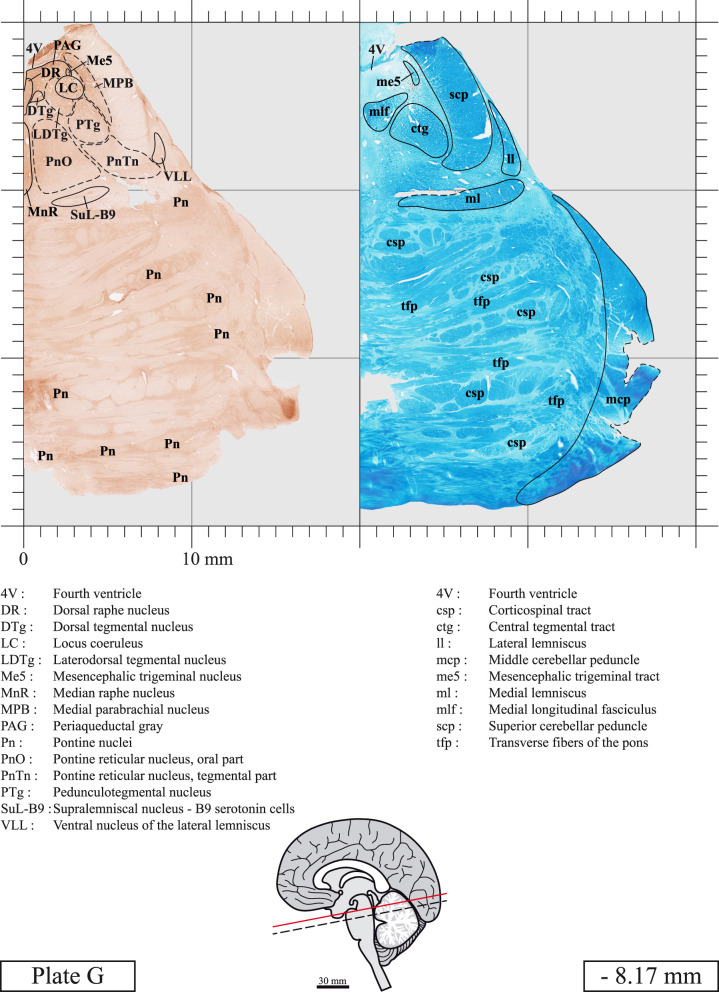

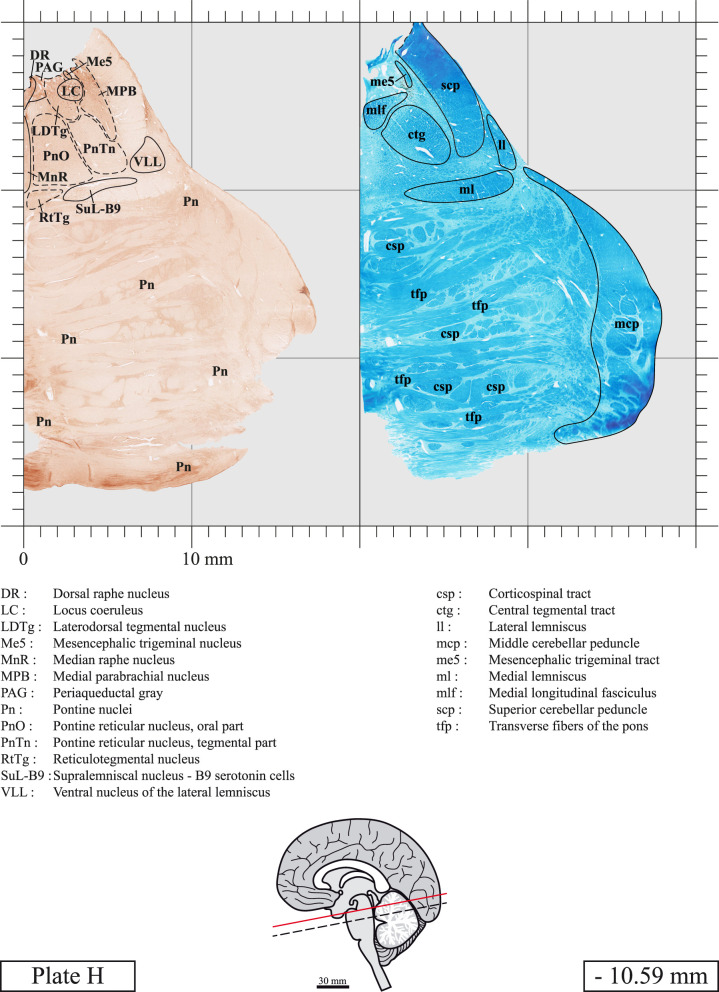











































































































## Data Availability Statement

The original contributions presented in the study are included in the article/Supplementary Material, further inquiries can be directed to the corresponding author.

## Ethics Statement

The studies involving human participants were reviewed and approved by Ethics Committee at Université Laval. The patients/participants provided their written informed consent to participate in this study.

## Author Contributions

MT and ÉP provided post-mortem human brain tissue. LG and MP were in charge of MRI and proceeded with brain dissection. MT, LG, and MP perfused and extracted the brain. LG cut the brainstem and stained sections with Cresyl Violet and Luxol Fast Blue. VC stained sections with ChAT and acquired and edited images. SS, AP, and MP were in charge of brainstem nuclei and fiber tracts segmentation. VC, AP, and MP wrote the manuscript. All authors contributed to the article and approved the submitted version.

## Conflict of Interest

The authors declare that the research was conducted in the absence of any commercial or financial relationships that could be construed as a potential conflict of interest.

## Publisher's Note

All claims expressed in this article are solely those of the authors and do not necessarily represent those of their affiliated organizations, or those of the publisher, the editors and the reviewers. Any product that may be evaluated in this article, or claim that may be made by its manufacturer, is not guaranteed or endorsed by the publisher.

## Supplementary Material

The Supplementary Material for this article can be found online: https://www.dropbox.com/sh/ln9zqv4dkygol2q/AAC80leqF0bYcI8ZLWERCIRXa?dl=0
